# Identification and ecotoxicity of the diclofenac transformation products formed by photolytic and photocatalytic processes

**DOI:** 10.1007/s11356-025-36466-5

**Published:** 2025-05-05

**Authors:** Enmanuel Cruz Muñoz, Giorgio Tseberlidis, Amin Hasan Husien, Simona Binetti, Fabio Gosetti

**Affiliations:** 1 Department of Earth and Environmental Sciences - DISAT, University of Milano-Bicocca, Piazza Della Scienza 1, 20126 Milano, Italy; 2 Department of Materials Science and Solar Energy Research Center (MIB-SOLAR), University of Milano-Bicocca, Via Cozzi 55, 20125 Milano, Italy; 3 Tecnologia E Sostenibilità per lo sviluppo dei Materiali Ceramici, CNR-ISSMC Istituto Di Scienza, Via Granarolo, 64, 48018 Faenza, RA Italy; 4 POLARIS Research Center, University of Milano-Bicocca, Piazza Della Scienza 1, 20126 Milano, Italy

**Keywords:** Diclofenac, Transformation products, Kesterite, Photodegradation, Mass spectrometry, Non-target analysis

## Abstract

Several studies were carried out to monitor and reduce the presence of diclofenac in the environment. Some of them reported an abatement of the drug of only 20–40%, whereas others based on advanced oxidation processes or photocatalytic methods mediated by TiO_2_ often highlighted high energy cost requirements and the formation of byproducts whose toxicity was higher than that of diclofenac. This study concerns both the photolytic and the photocatalytic degradation of diclofenac in aqueous media to evaluate its partial or total elimination and the formation of the transformation products, whose toxicity has been investigated. For this purpose, an ultra-high-performance liquid chromatography coupled with a high-resolution quadrupole time-of-flight-mass spectrometry method was used to elucidate the transformation products following a non-targeted approach based on mass data-independent acquisition. Twelve different transformation products were identified, two of them (TP1 and TP4) for the first time, and accordingly elucidated. Based on the accurate tandem mass spectrometry information acquired, a photodegradation mechanism was proposed. A toxicity assessment of the photoproducts was performed by in silico method. Although UV–vis photolysis degradation alone can abate the diclofenac after 120 min of irradiation, the formed transformation products persist at the end of the degradation, and they are mainly carbazole derivatives with comparable toxicity and similar persistence in the environment to that of diclofenac. On the contrary, the use of quaternary chalcogenide nanoparticles (Cu_2_ZnSnS_4_) is a successfully promising process for the drug photodegradation, resulting in the elimination of diclofenac and the formation of less toxic products than diclofenac at the end of degradation.

**Supplementary Information:**

The online version contains supplementary material available at 10.1007/s11356-025-36466-5.

## Introduction

The environmental impact of the growing worldwide consumption of pharmaceuticals is often undervalued. Once the active substance has achieved its intended purpose, it reaches the environment through a series of pathways that give it a second life causing potentially harmful effects. One of these pathways is related to metabolism, since the human organism does not completely degrade most pharmaceutically active substances, and releases metabolites and unaltered active substances through urine. Wastewater treatment plants (WWTP) are not capable of completely eliminating these compounds, which end up in ecosystems (Bound and Voulvoulis 2005). In 2005, a survey was conducted in the UK, in which it was estimated that 63.2% of unused or expired drugs ended up in household waste, 21.8% were returned to distribution centers and pharmacies, and 11.5% were dumped directly into the sink or toilets (Bound and Voulvoulis 2005). The average annual per capita consumption of drugs globally is estimated at 15 g, while in industrialized countries the value tends to be considerably higher, reaching a consumption of 50–150 g (Ternes and Joss 2006). Most of the molecules, known as emerging micropollutants (EMs), released into the environment are often of pharmaceutical origin, but they may also come from personal care products, industrial chemicals, or agricultural activities. In particular, the concentration of drug compounds in the environment and biological matrices ultimately depends on the frequent use and may range from micrograms per liter to nanograms per liter (Diaz-Camal et al. 2022). Therefore, analytical methods with low detection limits are required for their determination (Chen et al. 2023; Cruz Muñoz et al. 2024; Zaki et al. 2023).

A special mention must be made to a specific class of drugs: non-steroidal anti-inflammatory drugs (NSAIDs), which are compounds widely used for the reduction of pain, inflammation, and prevention of blood clot formation (Sobhani et al. 2023). Among all NSAIDs available on the market, diclofenac (DCF) is especially garnering popularity for the treatment of musculoskeletal and systemic inflammatory conditions. The estimated global consumption of this drug is 940 tons per year (Zhang et al. 2008).

The first degradation of DCF is due to transformations undergone in the metabolic process following absorption of the substance through the skin or orally. Orally taken up DCF is mostly eliminated following biotransformation into several metabolites and excreted mainly through urine (65%) (Davies and Andersen 1997), while the excretions have less than 1% of unmetabolized DCF and about 11% of DCF conjugated with glucuronic acid, taurine, and other molecules. In addition, there is evidence that the application of gels is followed by absorption of the substance of only about 6–7%, with the remainder either reaching the environment as a result of washing the skin or clothing or degrading due to sunlight (Davies and Andersen 1997). Although it is important to consider that metabolites may also turn out to be micropollutants, the most critical situation is due to that percentage of DCF that is released into the environment without having undergone any modification; in particular, reference is made to the DCF contained largely in water coming from urban uses and hospital facilities. From a structural point of view, the aromatic rings and the Cl atoms in the molecule give DCF high toxicity and a high degree of persistence and accumulation at the ecosystem level. For this reason, in 2013, the European Parliament and the Council of the European Union issued a new directive including DCF in the list of priority substances to control and monitor in the field of water policy (Directive 2013/39/EU). In this document, the maximum concentration limits in fresh and marine waters were defined, corresponding to 0.1 μg L^−1^ and 0.01 μg L^−1^, respectively.

DCF is difficult to remove from water through common purification methods. The efficiency of these varies depending on the technique used, but on average it is between 20 and 40%: a considerable amount of DCF remains in the water, as it leaves the WWTPs. However, an example of successful removal of DCF is found in the advanced oxidation process (AOP) method, where a total mineralization of DCF was achieved after 100 min of exposure to light (Pérez-Estrada et al. 2005). Nevertheless, a limitation of AOP and other complex treatments is the formation of intermediates whose toxicity could be similar to or greater than that of DCF, in addition to the fact they are carried out on a laboratory scale and have high-energy costs. Photocatalytic methods for DCF degradation were reported and they are mainly based on titanium dioxide (TiO_2_) as a photocatalyst due to its photochemical and thermal stability (Calza et al. 2006; Diaz-Angulo et al. 2019; Murgolo et al. 2018; Sarasidis et al. 2014). Recently, alternatives to the use of this type of catalyst have been found in the literature (Li et al. 2018; Liu et al. 2022; Silvestri et al 2019; Zhou et al. 2018). The implementation of more sophisticated and sensitive analytical techniques has allowed a better understanding of the DCF photo-degradative pathway (Luongo et al. 2021; Salgado et al. 2013) and, regardless of the different conditions used, a common problem arises: many products formed in this process are toxic and able to bioaccumulate in aquatic environments and are more difficult to remove than DCF (Diniz et al. 2015; Kolakovic et al. 2022).

Preliminary experiments in our laboratory demonstrated the effectiveness of using both UV photolysis irradiation and photocatalytic one mediated by kesterite nanoparticles (CZTS) for the abatement of DCF in aqueous media (Tseberlidis et al. 2024). Kesterite (Cu_2_ZnSnS_4_) is a quaternary chalcogenide with interesting applications in the field of photocatalysis. CZTS shows a high absorption coefficient in the visible and is nontoxic, insoluble, and stable in water, with an excellent ability to disperse homogeneously in solution when synthesized in nanoparticle form (Ito 2014; Trifiletti et al. 2019; Tseberlidis et al. 2020, 2021). To our knowledge, CZTS nanoparticles have never been tested for the degradation of EMs in water such as DCF, and some studies reported only their use for the photocatalytic degradation of some dyes (Apostolopoulou et al. 2018; Burhanuz Zaman et al. 2019; Henríquez et al. 2023; Hou et al. 2014; Phaltane et al. 2017; Semalti et al. 2021; Wei et al. 2019).

Therefore, the aim of this study is the characterization of DCF transformation products (TPs) after photolysis and catalytic photodegradation in water by an ultra-high performance liquid chromatography quadrupole time-of-flight tandem mass spectrometry (UHPLC-QTOF MS/MS) method. For this purpose, four laboratory experiments were carried out, considering the use or not of ultraviolet (UV) radiation and the use of CZTS, finally proposing a photodegradation pathway of DCF and the toxicity assessment of DCF TPs by in silico predictions.

## Material and methods

### Reagents and standard solutions

DCF sodium salt (≥ 98.5%) was purchased from Sigma-Aldrich (St.Louis, USA), whereas water (UHPLC-MS grade), methanol (UHPLC-MS grade), acetonitrile (UHPLC-MS grade), ammonium acetate (LC–MS grade), and formic acid (LC–MS grade) were acquired from Carlo Erba (Milan, Italy). All chemical reagents used for the CZTS Nanoparticles synthesis were purchased from Sigma-Aldrich Corporation (St. Louis, MO, USA) and used without any further purification: copper (II) acetate monohydrate (Cu(CH_3_COO)_2_ · H_2_O, > 99%), zinc (II) acetate dihydrate (Zn(CH_3_COO)_2_ · 2H_2_O, 99.99%), sulfur powder, > 99.5%), tin (II) chloride dihydrate (SnCl_2_ · 2H_2_O, > 98%), Oleylamine (OLA, 98%).

Fifteen standard solutions were prepared for DCF quantification. First, a DCF standard stock solution (1000 mg L^−1^) in ultrapure water was prepared, from which a working solution was prepared (10 mg L^−1^) in methanol. Finally, this solution was used to prepare the standards ranging from 0.05 to 500 μg L^−1^ by appropriate dilution in the mobile phase at initial gradient conditions.

### Instrumentation

An LSB530 xenon lamp (300 W) with a color temperature ranging from 6050 to 6350 K was acquired from Quantum Design (Roma, Italy) and used for the photodegradation experiments. It was equipped with an IR and UV filter, used in experiments involving irradiation with visible light only. The working power during the experiments was 216 W and it was used out of focus to guarantee an irradiance equal to 1000 W/cm^2^ at the usage distance, in order to emulate 1 sun (AM1.5G) irradiance at laboratory scale.

The UHPLC analyses were carried out by ACQUITY UPLC H-Class system (Waters Corporation, Milford, USA) coupled with the Xevo G2-XS QTof Mass Spectrometer (Waters Corp., Milford, MA, USA), using electrospray ionization (ESI) as an ion source. MassLynx 4.2 software was used for instrument control and data acquisition (Waters Corporation, Milford, USA) whereas data processing operations were carried out by MS-Dial (ver. 5.1.230517) and MS-Finder (ver.3.52) (Tsugawa et al. 2015, 2016).

### Chromatographic and mass spectrometric conditions

An Acquity HSST3 C18 column (100 × 2.1 mm, 1.8 μm; Water Corporation, Milford, USA) was used as stationary phase, whereas the mobile phase was a mixture of 1.0 mM aqueous ammonium acetate/methanol 98/2 v/v (solvent A) and 1.0 mM aqueous ammonium acetate (1 mM)/methanol 2/98 v/v (solvent B). The mobile phase flow rate was 0.300 mL min^−1^ eluting in gradient mode as follows: 0.0–0.5 min, 5% solvent B; 0.5–6.0 min, 100% solvent B; 6.0–7.0 min, 100% solvent B. The injection volume was 5 μL and the column oven temperature was set at 40 °C.

ESI operated in both positive and negative ionizations. Accurate mass data were collected in the data-independent acquisition MS^E^ mode by alternating low and high energy applied to the collision cell; i.e., in the low-energy MS mode, data were collected at a constant collision energy of 6 V, whereas in high-energy mode, the collision energy was increased from 15 to 30 V with a scan time of 0.1 s.

Spectra were recorded in the mass range of *m/z* 50–650. The source parameters were set as follows: electrospray capillary voltage 2.50 kV, sampling cone 10 V, and source and desolvation temperatures 140 °C and 600 °C, respectively. The cone and desolvation gas flows were 0 and 1000 L/h, respectively. A resolving power of 30,000 was applied both in full and in MS/MS scan modes. The mass spectrometer was calibrated with sodium formate (0.5 mM in a solution acetonitrile/water 80/20 v/v) and mass correction was performed by using a known mixture as LockMass, which consists of *leucine-enkephalin* (200 pg mL^−1^) and *GFP [Glu*^*1*^*]-Fibrinopeptide B* (100 fmol μL^−1^) in 50% acetonitrile and 0.1% formic acid. This mixture was infused at 8 μL min^−1^ and acquired every 30 s.

### Synthesis and characterization of CZTS nanoparticles

**Fig. 1:**
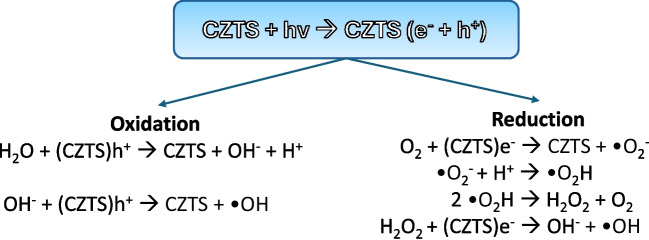
Oxidation and reduction reactions undergone by exposure of CZTS to light radiation, with the consequent generation of radicals

Kesterite possesses the properties of a p-type semiconductor and possesses a band-gap that can vary between 1.4 and 1.6 eV, depending on the number of copper vacancies, the order degree of the crystal lattice, and the morphology of the material (Ito 2014; Trifiletti et al.^2019^; Tseberlidis et al. 2020, 2021). This band-gap value allows it to exploit virtually the entire range of visible light to promote electrons from the valence band to the conduction band. Thus, highly reactive free radicals are generated according to the reaction pattern shown in Fig. 1.

Moreover, it consists of naturally abundant elements and is therefore easy to produce in large quantities and at low cost.

Nanoparticles were synthesized by following the *hot-injection* procedure, where a cold solution containing the precursors is poured into a hot solution consisting of a surfactant and a solvent with a high boiling point. The precursors used were copper acetate hydrate (Cu(CH_3_COO)_2_·H_2_O), zinc acetate dihydrate (Zn(CH_3_COO)_2_·2H_2_O), tin chloride dihydrate (SnCl_2_·2H_2_O), and elemental sulfur. The binder used was oleylamine (Hasan Husien et al. 2025; Khanzada et al. 2016).

Precursors and oleylamine were placed in a flask equipped with a reflux condenser in an anoxic environment. The solution was kept at 150 °C for 1 h and the temperature was raised to 200–280° C. A solution of sulfur powder in oleylamine, degassed in the anoxic environment for 1 h at 60 °C, was then rapidly injected into the reaction medium and the temperature was kept constant at 280 °C for 30 min, after which it was then allowed to cool down to room temperature. Afterwards, the solution was washed with a solution chloroform/ethanol 1/5 (v/v) and centrifuged at 8000 rpm for 2 min. The supernatant was removed, and then the washing process was repeated several times to remove any residual oleylamine. For the characterization of CZTS nanoparticles, scanning electron microscopy analyses (SEM) using the Tescan VEGA TS5136XM (Brno, Czech Republic) and transmission electron microscopy (TEM) investigations using a JEOL JEM-2100PLUS (Tokyo, Japan) with an emission voltage of 200 kV were performed. Fig. S1 of Supplementary Materials shows the SEM and TEM images of synthesized CZTS nanoparticles.

### Photodegradation experiments

Four sets of experiments were conducted, i.e., photolysis with visible radiation (P Vis), photolysis with ultraviolet and visible radiation (P UV–Vis), photocatalysis mediated by CZTS nanoparticles with ultraviolet and visible radiation (PC UV–Vis), and photocatalysis mediated by CZTS nanoparticles with visible radiation (PC Vis). For the photocatalysis experiments, 28 mg of CZTS was weighed in a vial and 25.2 mL of milli-Q water was then added. The resulting suspension was magnetically stirred for 10 min to disperse homogenously the CZTS nanoparticles and, finally, 2.8 mL of DCF stock solution (100 mg L^−1^) was added to operate at 10 mg L^−1^ DCF concentration. The vial was finally closed by using a rubber stopper with a needle to avoid the accumulation of hydrogen that could be generated inside the vial. The vessel was placed at 0.5 m from the xenon lamp and kept under stirring for 2 h. Aliquots of 2.5 mL each of independent solutions were withdrawn at different photodegradation times (0, 1, 3, 5, 15, 30, 60, 120 min), indicated as *t*_*0*_ to *t*_*7*_ hereinafter. Each aliquot was filtered with a hydrophilic 0.22 μm PTFE filter (Carlo Erba, Milano, Italy) and kept at − 20 °C until UHPLC-MS/MS analysis. Before introducing and filling another DCF solution at *t*_*0*_ into the cell, the latter was emptied and carefully cleaned. The procedure for the photolytic experiments was the same, except for the use of the catalyst. For the analysis of the samples resulting from each set of photodegradation experiments (from *t*_*0*_ to *t*_*7*_, 32 samples in total), 10 μL was withdrawn and diluted with 190 μL of mobile phase (solvent A). Each analysis was repeated three times.

### Chemical oxygen demand

Chemical oxygen demand was determined according to the normative ISO 15705:2002 (ISO 15705:2002 2002).

To understand the extent of the mineralization during the three different photodegradation experiments (P UV–Vis, PC UV–Vis, and PC Vis), COD determinations were carried out.

The experiments were performed at *t*_*0*_ (before irradiation) and *t*_*7*_ (end of degradation) for all three sets of experiments. The extent of degradation was calculated as the percentage ratio between the difference of COD value (initial COD minus final COD value) and initial COD value.

### Toxicity assessment

Environmental impact and the toxicity of TPs formed during the different irradiation experiments were predicted in silico using quantitative structure–activity relationships (QSARs) and read-across models using the free available internet resources Toxicity Estimation Software Tool (T.E.S.T) (U.S. Environmental Protection Agency, 2020) and VEGA software (Benfenati et al. 2013).

T.E.S.T. software was used to predict the acute aquatic toxicity in fish (LC50-96 h), *Daphnia magna* (LC50-48 h), *Tetrahymena pyriformis* (IGC50-48 h), and the acute oral toxicity in rat (LD50) after using the Simplified Molecular Input Line Entry System (SMILES) notation as input and following the consensus method and relaxing the fragment constraint in all cases.

VEGA software was used to predict the mutagenicity (consensus method), developmental toxicity, bioaccumulation, ready biodegradability, and persistence in water, soil, and sediments, also using the SMILES notation.

## Results and discussion

### Photodegradation of DCF and UHPLC-MS/MS analysis

**Fig. 2:**
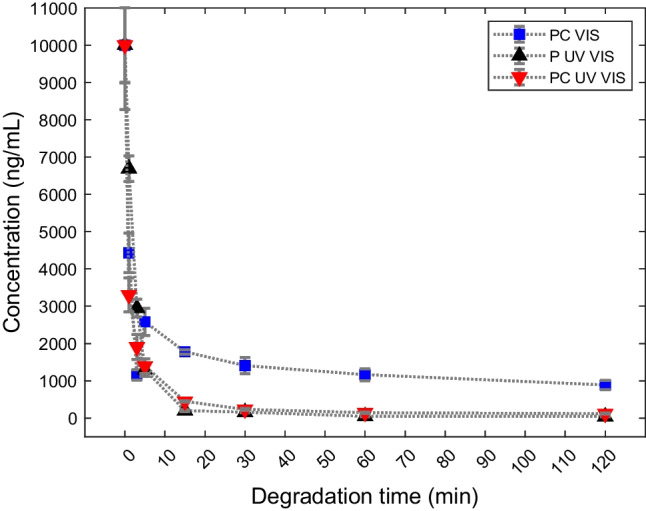
Trend of DCF concentration (ng/mL) as a function of degradation time of PC VIS experiment (blue square), P UV VIS (black triangle) and PC UV VIS (red triangle)

DCF degrades very rapidly forming TPs in all irradiation experiments (Fig. 2), except for the P Vis experiment where no evidence of degradation was found. Table 1 shows the removal percentage of DCF from the aqueous solution after 1, 5, and 120 min of irradiation. The final solutions at 120 min of all the photodegradation experiments were still subjected to irradiation up to a time of 240 min, and no change in the intensities of DCF and its TP formed was highlighted.

**Table 1:** Percentage of DCF degradation based on chromatographic peak area diminution with respect to *t*_*0*_ (*t*_*1*_ = 5 min, *t*_*3*_ = 5 min, *t*_*7*_ = 120 min)

Experiment	% DCF removal at *t*_*1*_	% DCF removal at *t*_*3*_	% DCF removal at *t*_*7*_
P UV–Vis	32.8	87.0	99.6
PC UV–Vis	70.7	87.6	99.0
PC Vis	55.6	74.2	91.1

It is observed that drug removal after 120 min is more than 90% in all three experiments. This percentage is higher than most of the common treatments used in sewage treatment plants, which are based on advanced and energy-consuming oxidation processes (Kodom et al. 2021; Yang et al. 2017; Zhu et al. 2024). Furthermore, it is observed that irradiation with UV–Vis light causes a higher removal rate compared to the use of only Vis radiation. A very high removal rate was already observed after 1 min in the PC UV–Vis experiments.

To identify potential TPs by UHPLC-MS/MS, MS-Dial software was used to make a project for each set of experiments including all the MS/MS data from *t*_*1*_ to *t*_*7*_, using *t*_*0*_ as a reference for peak alignment. For the reprocessing of the acquired data, 0.1 min for the retention time and 0.015 Da for the *m/z* were imposed as tolerance parameters for the peak alignment. Moreover, to consider a candidate as a TP, the ratio of the sample peak area to that of the reference (*t*_0_) must be greater than 10 times. Then, a chromatographic peak list was created including all the possible m/z candidates.

In addition, MS-finder software was used for the characterization of the chemical structures of the identified TPs.

### UV–Vis photolysis experiments (P UV–Vis)

In this experiment, five TPs were identified (TP1, TP2, TP3, TP4, and TP5), as shown in the chromatogram of Fig. S2 (Supplementary Material), where the extracted ion chromatogram (XIC) of DCF and each TP was plotted, min–max scaling the signals to better compare them visually. The TP evolution profiles are shown in Fig. S3 (Supplementary Material).

TP1 reaches its maximum concentration almost immediately after DCF is exposed to light, whereas TP2 and TP3 after 3 min of irradiation, and all remain in solution at the end of irradiation (Fig. S3). TP4 reaches its maximum concentration at the end of the irradiation experiment, and TP5 reaches its maximum concentration after only 1 min of DCF being irradiated, completely disappearing after 15 min.

### UV–Vis photocatalysis experiments

**Fig. 3:**
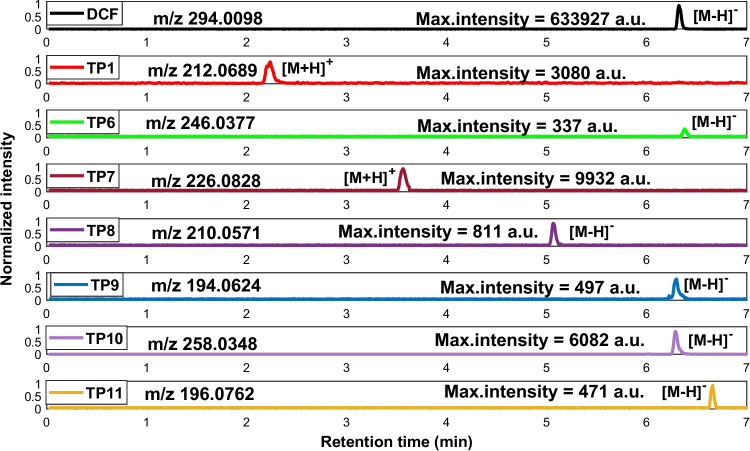
Extracted ion chromatogram of DCF (black trace) and seven TPs (color traces) formed in the UV–Vis photocatalysis experiments. The chromatographic peak of each TP was selected at the degradation time of its maximum intensity. All the peak intensities are min–max scaled

Figure 3 shows the chromatographic peaks of the seven TPs identified in this experiment, (TP1, TP6, TP7, TP8, TP9, TP10, and TP11), of which TP1 was already identified in the P UV–Vis.

The evolution profiles of the TPs identified are shown in Fig. S4 (Supplementary Material). TP6, TP8, and TP9 reach their maximum concentration after 5 min, TP10 after 3 min, and TP11 after 60 min from the start of irradiation, and they gradually decrease until completely disappearing at 120 min.

### Vis photocatalysis experiments

Three TPs were identified (TP1, TP6, and TP12) and shown in Fig. S5 (Supplementary Material). TP1 was previously identified in the P UV–Vis and PC UV–Vis experiments, whereas TP6 was identified in the PC UV–Vis ones.

After 60 min, TP12 reaches its maximum concentration, remaining still in the solution at the end of radiation exposure (Fig. S6).

### Identification of the TPs

Chemical structure elucidation of TPs was based on the interpretation of the high-accurate and high-resolution MS/MS acquired spectra, considering the molecular formula within the average MS accuracy obtained around 10 ppm, the relative abundance of the isotopic cluster (tolerance 10%), and the number of rings and double bonds (RDBs). All the related information are summarized in Table 2. All MS/MS spectra of DCF and TPs (from TP1 to TP12) with the identification of the most abundant m/z signals are reported in Figs. S7-S2 (Supplementary Material), respectively.

**Table 2.**
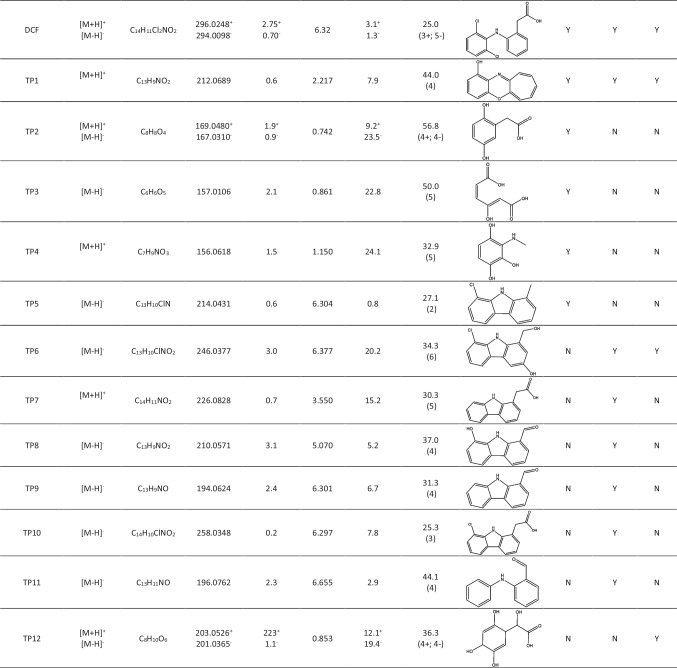
Elemental composition, accurate m/z, isotopic tolerance, retention time, mass error, average error for the tandem mass spectrometry (MS/MS) data calculated through the number of the product ions identified in ESI+ or ESI- and reported in parenthesis, proposed chemical structure of the TP formed and type of irradiation experiment

Overall, in the three irradiation experiments, 12 TPs were found and their chemical structures elucidated, two of which (TP1 and TP4) have never been reported, to our knowledge, in the literature yet.

The proposed structure of TP1 involves the loss of the carboxyl group (-COOH) and a rearrangement of the aromatic ring leading to the formation of a 7-carbon cycle. On the other ring, the attack of OH^·^ leads to the substitution of the atoms of chlorine with the hydroxyl group and the subsequent formation of a new C-O bond. TP1 has been also found in the other two sets of degradation experiments, as shown in Fig. S2 and Fig. S5 (Supplementary Material).

TP2 formation is probably due to the cleavage of the C-N bond with the formation of a reaction intermediate that is subsequently hydroxylated by OH^·^ radicals (Pérez-Estrada et al. 2005). However, the bonding position on the aromatic ring cannot be precisely determined only by mass spectrometry analysis. Nevertheless, it is assumed the ortho- and meta-hydroxylation with respect to the aliphatic chain, where this structure was verified by MS/MS spectrum measurements and NMR spectroscopy studies concerning the oxidation of DCF in water using sodium hypochlorite (Luongo et al. 2021).

The possible mechanism of TP3 formation involves the cleavage of the C-N bond, the hydroxylation (which can occur indifferently before or after the cleavage of the C-N bond), and the opening of the benzene ring, as reported by Salaeh et al. (2016) in a study concerning the removal of DCF using TiO_2_-based zeolite as photocatalyst.

The proposed structure of TP4 is based on the loss of the carboxyl group, followed by hydroxylation and cleavage of the C–C bond, resulting in the loss of the aromatic ring.

The proposed structure of TP5 involves a loss of a chloride ion with the formation of 8-chloro-9H-carbazol-1-yl acetic acid. The subsequent loss of the carboxyl group leads to the formation of the final product (Alharbi et al. 2017; Murgolo et al. 2018).

The formation of TP6 is due to an initial DCF hydroxylation on the aromatic ring. The reaction proceeds with the loss of a chloride ion and the subsequent cyclization with a carbazole formation. Lastly, the loss of the -CO group leads to the formation of the final product (Murgolo et al. 2018).

The proposed structure of TP7 was previously found in studies focused on the evaluation of the DCF toxicological effects in the environment (Ellepola et al. 2022) and the photocatalytic removal of DCF using an Ag_3_PO_4_/GCN-impregnated MIL-88 catalyst (Hemkumar et al. 2023), a diphenyl catalyst of reduced graphitic carbon nitride (Jin et al. 2020), and a g-C_3_N_4_ nanotube catalyst (Jiménez-Salcedo et al. 2021). The formation mechanism involves double loss of HCl followed by carbazole formation.

The formation mechanism of TP8 involves the loss of chlorine from DCF with carbazole formation. The attack of a hydroxyl radical leads to the replacement of the other chlorine atom and at last the loss of the carboxylic group and the oxidation to form an aldehyde (Wang et al. 2023). TP9 forms with the same mechanism as TP8, but with a further loss of the hydroxyl group on the aromatic ring (Wu et al. 2018).

The proposed structure of TP10 has been found in many studies concerning the removal and degradation of DCF in aqueous solutions using different catalysts such as hydroxyapatite-TiO_2_ (Murgolo et al. 2018), TiO_2_-based zeolite (Salaeh et al. 2016), immobilized TiO_2_ (Di Credico et al. 2015), palladium quantum dots (Liu et al. 2022), Ag_3_PO_4_/GCN-impregnated MIL-88 (Hemkumar et al. 2023), defect-modified reduced graphitic carbon nitride (Jin et al. 2020), g-C_3_N_4_ nanotubes (Jiménez-Salcedo et al. 2021), manganese oxide (Wu et al. 2018), Fe nanoclusters anchored to g-C_3_N_4_ rods (Li et al. 2022), and ZnO (Meroni et al. 2021). The proposed mechanism for the formation of TP10 consists of possible photocyclization, with loss of HCl and the formation of a carbazole, that turns out to be an intermediate for the formation of additional degradation products derived from the loss of the carboxyl group (Alharbi et al. 2017; Jiménez-Salcedo et al. 2021; Wang et al. 2023; Wu et al. 2018) to give TP5, or a further loss of HCl to form TP7.

As concerns TP11, the formation process is probably due to an initial double loss of chlorine followed by the formation of an intermediate that undergoes decarboxylation and hydroxyl radical attack leading to the formation of the final product. The structure may undergo other degradation processes, such as C-N bond breaking and further radical attack to form low molecular weight structures (Boukhatem et al. 2017; Nguyen et al. 2020).

The proposed chemical structure for TP12 is based on an initial attack of DCF by the OH^·^ radical. Subsequently, the loss of chlorine and scission of the C-N bond leads to the formation of the proposed fragment. The same TP was already reported for the degradation of DCF in an aqueous solution by a Cold Atmospheric Plasma AOP method (Kumar et al. 2023).

**Fig. 4:**
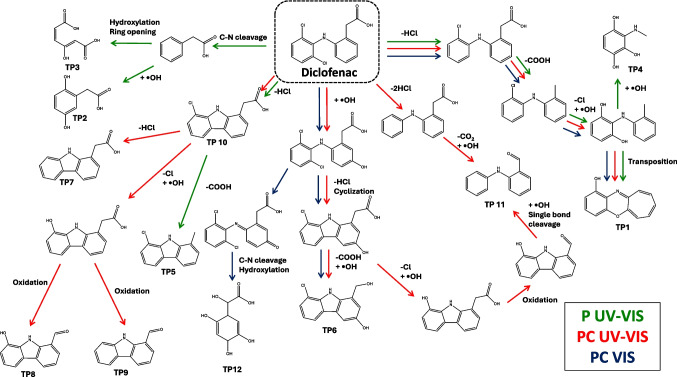
Overall photodegradation pathway of DCF. The TPs formed in the 3 different experiments are reported: P UV–Vis (green), PC UV–Vis (red), and PC Vis (blue)

Based on the proposed structures and mechanisms hypothesized and verified also through literature search, the possible pathway of DCF degradation is depicted in Fig. 4.

The COD data confirmed those obtained with UHPLC-MS/MS analysis. The best degradation extent was obtained by PC-UV–Vis with 90.4%, then 85.3% for PC Vis, and at last only 23.6% for P UV–Vis, confirming an incomplete degradation of TPs formed.

### In silico assessment of TP toxicity

Predicted values of toxicity using the Toxicity Estimation Software Tool (TEST 2024) and VEGA software (VEGA QSAR 2024) are reported in Table 3.

**Table 3:** In silico prediction of ecotoxicity, mutagenicity, bioaccumulation, and persistence of diclofenac and its TPs by T.E.S.T and VEGA software

ID	ToxFish^1^	ToxDaph^2^	ToxtPyr^3^	ToxRat^4^	Ames^5^	ToxDev^6^	Biodeg^7^	BCFmAG^8^	PerWat^9^	PerSed^10^	PerSoil^11^
DCF	0.5	3.5	1.5	244.0	NON-Mutagenic	Toxicant (MR)	nonRB	3.12	0	49	40
TP1	1.0	9.1	3.4	1049.3	Mutagenic	Toxicant (LR)	nonRB	2.28	8	156	41
TP2	37.7	17.0	160.3	212.5	NON-Mutagenic	Toxicant (GR)	PRB	0.03	4	49	5
TP3	105.3	145.3	1451.4	5138.3	NON-Mutagenic	NON-Toxicant (GR)	PRB	− 0.04	5	23	11
TP4	49.3	14.8	109.3	642.9	NON-Mutagenic	NON-Toxicant (MR)	-	− 0.05	2	49	7
TP5	0.2	0.7	4.9	609.3	Mutagenic	Toxicant (LR)	PnonRB	2.98	0	49	23
TP6	0.6	2.5	706	1335.7	Mutagenic	Toxicant (LR)	PnonRB	1.03	0	49	40
TP7	0.8	6.4	10.7	3586.4	Mutagenic	Toxicant (GR)	PRB	1.64	0	49	5
TP8	0.4	2.6	6.4	635.8	Mutagenic	Toxicant (LR)	PRB	1.33	4	49	5
TP9	0.5	2.6	7.7	1645.3	Mutagenic	Toxicant (LR)	PRB	1.92	4	49	5
TP10	0.4	2.6	4.6	2170.8	Mutagenic	Toxicant (MR)	PnonRB	2.20	0	49	40
TP11	1.7	9.2	9.6	1396.7	NON-Mutagenic	Toxicant (LR)	nonRB	1.88	4	49	5
TP12	536.3	280.3	-	11,511.3	NON-Mutagenic	NON-Toxicant (GR)	PRB	− 0.05	10	23	16

^1^Acute Toxicity Fish (Fathead minnow LC50 (96 h)). CONSENSUS model T.E.S.T., mg/L

^2^*Daphnia magna* LC50 (48 h). CONSENSUS model T.E.S.T., mg/L

^3^* T. pyriformis* IGC50 (48 h). CONSENSUS model T.E.S.T., mg/L

^4^Oral rat LD50. CONSENSUS model T.E.S.T., mg/kg

^5^Mutagenicity (Ames test) CONSENSUS model (version 1.0.4), VEGA

^6^Developmental Toxicity model (CAESAR) (version 2.1.8), VEGA

^7^Ready Biodegradability model (IRFMN) (version 1.0.10), VEGA

^8^BCF model (Arnot-Gobas) (version 1.0.1), VEGA, log(BCF)

^9^Persistence (water) quantitative model (IRFMN) (version 1.0.1), VEGA, days

^10^Persistence (sediment) quantitative model (IRFMN) (version 1.0.1), VEGA, days

^11^Persistence (soil) quantitative model (IRFMN) (version 1.0.1), VEGA, days

^*^PnonRB possible non-readily biodegradable, *PRB* possible readily biodegradable, *nonRB* non-readily biodegradable, *LR* low reliability, *MR* medium reliability, *GR* good reliability

**Fig. 5:**
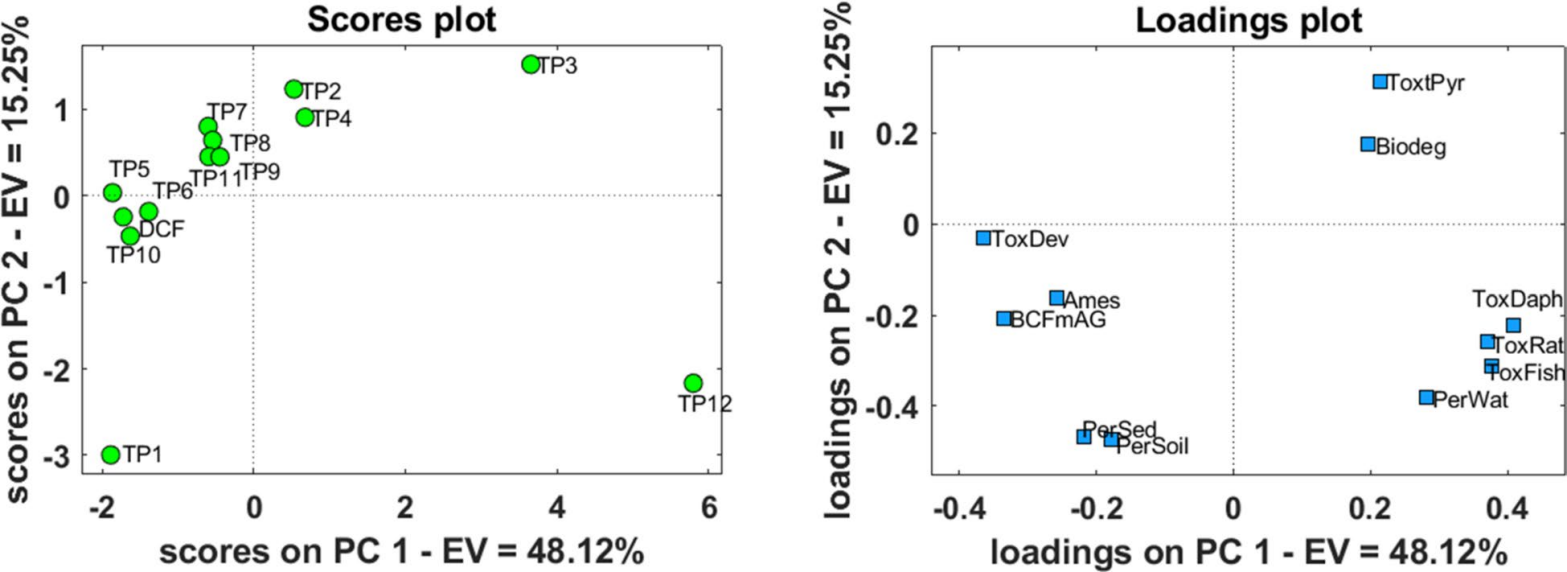
Principal component analysis (PCA) results of the toxicological and environmental fate endpoints for DCF ant its TPs: **a** score plot and **b** loading plot of the first two PCs. The variable labels in the loading plot are given in the Table 3

Afterwards, an exploratory analysis of the data was performed using principal component analysis (PCA), whose scores and loadings plots for the first two principal components (PCs) are reported in Fig. 5a and b, respectively. The projection of the predicted values in the orthogonal space leads to 63.4% of the total data variance explained in the first two PCs and 21.5% in the next two PCs. PC1 highlights differences between the samples TP3 and TP12 (positive scores), which are characterized by low toxicity values, from the samples TP1, TP5, TP6, and DCF (negative scores), which are characterized by developmental toxicity and bioaccumulation as the most characteristic variables. PC2 mainly highlights the differences in terms of persistence in sediments and soil (negative loadings) versus *T. pyriformis* (positive loadings). Indeed, TP1 which has negative scores in PC2 has the highest sedimentary and soil persistence predicted value, whereas TP2 (positive score values in PC2) shows the lowest persistence in sediments.

According to the Globally Harmonized System (GHS) of classification and labelling of chemicals (United Nations 2021), DCF, TP1, TP5, TP6, TP7, TP8, TP9, and TP10 are predicted as moderately toxic for aquatic environments and classified into the Category Acute 1 (96 h LC50 ≤ 1 mg L^−1^), whereas TP11 is classified into the Category Acute 2 (96 h LC50 > 1 mg L^−1^ but ≤ 10 mg L^−1^), and TP2 and TP4 classified as Category Acute 3 (96 h LC50 > 10 mg L^−1^ but ≤ 100 mg L^−1^). TP3 and TP12 are excluded from the moderately toxic for aquatic environments classification due to values exceeding the threshold. Considering the toxicity in rats by oral ingestion, DCF, and TP2 are labelled as Category 3 (LD50 > 50 mg kg^−1^ but ≤ 300 mg kg^−1^), while TP1, TP4, TP5, TP6, TP8, TP9, and TP11 are classified as Category 4 (LD50 > 300 mg kg^−1^ but ≤ 2000 mg kg^−1^), being TP3, TP7, TP10, and TP12 classified as Category 5 (LD50 < 2000 mg kg^−1^ but ≤ 5000 mg kg^−1^). In addition, most TPs were predicted as interfering potential for the normal development of humans or animals, with the exceptions of TP3, TP4, and TP12. According to REACH regulation EC No 1907/2006, all of them have a low potential for bioaccumulation (logBFC < 3.3).

## Conclusions

The PC UV–Vis experiments produced the largest number of TPs (7 in total). About 70% of these products are carbazoles that are formed by photocyclization with loss of the Cl atoms and subsequent oxidation. The presence of UV–Vis radiation and CZTS nanoparticles seems to proceed with the degradation reaction even toward these products whose toxicity for aquatic environments is similar to that of DCF, while the toxicity by oral ingestion is lower. However, about 80% of the carbazoles found are formed over time and then degrade and disappear after 120 min of irradiation. On the contrary, in the PC Vis and P UV–Vis experiments, a low percentage of carbazoles (30% and 20%, respectively) were formed, while molecules derived from the cleavage of the C-N bond are generated which, after the attack of hydroxyl radicals, form phenylacetic acid derivatives. These molecules are less toxic than DCF and derived carbazoles but are not completely removed from the solution after 120 min in most cases.

Based on the results obtained, the most effective removal of DCF occurs in the photocatalysis experiments with UV–Vis light, where a mineralization of 90.4% is reached. The use of CZTS nanoparticles, therefore, may be a valuable catalyst for the removal of DCF and its TPs in water. The elucidation of some of the TPs obtained during the irradiation experiments was successfully carried out by following a high-resolution MS/MS data analysis approach.

Therefore, the efficacy of CZTS for the removal of DCF compared to some commonly used WWTP removal treatments appears to be promising. It has been observed how the catalytic activity of CZTS influences the degradation of DCF, and this may vary depending on the microscopic treatments after its synthesis or simply on the synthesis method. Consequently, further research and development of methods that improve the synthesis of CZTS particles by maximizing their catalytic activity is needed. The applicability of CZTS in the environment lies in its non-toxicity, its low cost compared to AOP methods, and the abundance of its elements. The behavior of nanoparticles in the presence of other micropollutants and in aqueous matrices from filtered surface water, where other chemical species, such as inorganic ions and organic molecules, may interact with the catalyst, will need to be further investigated. Finally, considering that the concentration of DCF used in the samples analyzed is several orders of magnitude higher than that found in the environment, the process will have to be optimized for applications in real conditions in future studies.

## Supplementary Information

Below is the link to the electronic supplementary material.

Supplementary file1 (PDF 3616 KB)

## Data Availability

All data supporting the findings of this study in its current form are available within the paper and supplied materials.

## References

[CR1] Alharbi SK, Kang J, Nghiem LD, Van de Merwe JP, Leusch FDL, Price WE (2017) Photolysis and UV/H_2_O_2_ of diclofenac, sulfamethoxazole, carbamazepine, and trimethoprim: identification of their major degradation products by ESI-LC-MS and assessment of the toxicity of reaction mixtures. Process Saf Environ Prot 112:222–234. 10.1016/j.psep.2017.07.015

[CR2] Apostolopoulou A, Mahajan S, Sharma R, Stathatos E (2018) Novel development of nanocrystalline kesterite Cu_2_ZnSnS_4_ thin film with high photocatalytic activity under visible light illumination. J Phys Chem Solids 112:37–42. 10.1016/j.jpcs.2017.09.005

[CR3] Benfenati E, Manganaro A, Gini G (2013) VEGA-QSAR: AI inside a platform for predictive toxicology. CEUR Workshop Proc 1107:21–28

[CR4] Boukhatem H, Khalaf H, Djouadi L, Marin Z, Navarro RM, Santaballa JA, Canle M (2017) Diclofenac degradation using mont-La (6%)-Cu_0.6_Cd_0.4_S as photocatalyst under NUV–Vis irradiation. Operational parameters, kinetics, and mechanism. J Environ Chem Eng 5:5636–5644. 10.1016/j.jece.2017.10.054

[CR5] Bound JP, Voulvoulis N (2005) Household disposal of pharmaceuticals as a pathway for aquatic contamination in the United Kingdom. Environ Health Perspect 113:1705–1711. 10.1289/ehp.8315 16330351 10.1289/ehp.8315 PMC1314909

[CR6] Burhanuz Zaman M, Mir RA, Poolla R (2019) Growth and properties of solvothermally derived highly crystalline Cu_2_ZnSnS_4_ nanoparticles for photocatalytic and electrocatalytic applications. Int J Hydrogen Energy 44:23023–23033. 10.1016/j.ijhydene.2019.07.026

[CR7] Calza P, Sakkas VA, Medana C, Baiocchi C, Dimou A, Pelizzetti E, Albanis T (2006) Photocatalytic degradation study of diclofenac over aqueous TiO_2_ suspensions. Appl Catal B Environ 67:197–205. 10.1016/j.apcatb.2006.04.021

[CR8] Chen H-W, Liu H-T, Kuo Y-N, Yang D-P, Ting T-T, Chen J-H, Chiu J-Y, Jair Y-C, Li H-C, Chiang P-J, Chen W-R, Lin M-C, Hsu Y-H, Chen P-S (2023) Rapid and sensitive dilute-and-shoot analysis using LC-MS-MS for identification of multi-class psychoactive substances in human urine. J Pharm Biomed Anal 233:115443. 10.1016/j.jpba.2023.115443 37210892 10.1016/j.jpba.2023.115443

[CR9] Cruz Muñoz E, Termopoli V, Orlandi M, Gosetti F (2024) Non-targeted identification of tianeptine photodegradation products in water samples by UHPLC-QTOF MS/MS. Chemosphere 361:142534. 10.1016/j.chemosphere.2024.142534 38849097 10.1016/j.chemosphere.2024.142534

[CR10] Davies NM, Andersen KE (1997) Clinical pharmacokinetics of diclofenac. Ther Insights Pitfalls Clin Pharmacokinet 33:184–213. 10.2165/00003088-199733030-00003 10.2165/00003088-199733030-00003 9314611

[CR11] Di Credico B, Bellobono IR, D’Arienzo M, Fumagalli D, Redaelli M, Scotti R, Morazzoni F (2015) Efficacy of the reactive oxygen species generated by immobilized TiO_2_ in the photocatalytic degradation of diclofenac. Int J Photoenergy 2015:1–13. 10.1155/2015/919217

[CR12] Diaz-Angulo J, Porras J, Mueses M, Torres-Palma RA, Hernandez-Ramirez A, Machuca-Martinez F (2019) Coupling of heterogeneous photocatalysis and photosensitized oxidation for diclofenac degradation: role of the oxidant species. J Photochem Photobiol A Chem 383:112015. 10.1016/j.jphotochem.2019.112015

[CR13] Diaz-Camal N, Cardoso-Vera JD, Islas-Flores H, Gómez-Oliván LM, Mejía-García A (2022) Consumption and occurrence of antidepressants (SSRIs) in pre- and post-COVID-19 pandemic, their environmental impact and innovative removal methods: a review. Sci Total Environ 829:154656. 10.1016/j.scitotenv.2022.154656 35318057 10.1016/j.scitotenv.2022.154656

[CR14] Diniz MS, Salgado R, Pereira VJ, Carvalho G, Oehmen A, Reis MAM, Noronha JP (2015) Ecotoxicity of ketoprofen, diclofenac, atenolol and their photolysis byproducts in zebrafish (*Danio rerio*). Sci Total Environ 505:282–289. 10.1016/j.scitotenv.2014.29.103 25461029 10.1016/j.scitotenv.2014.09.103

[CR15] Directive 2013/39/EU of the European parliament and of the council amending directives 2000/60/EC and 2008/105/EC as regards priority substances in the field of water policy. https://eur-lex.europa.eu/LexUriServ/LexUriServ.do?uri=OJ:L:2013:226:0001:0017:en:PDF. Accessed 2 May 2025

[CR16] Ellepola N, Viera T, Patidar PL, Rubasinghege G (2022) Fate, transformation and toxicological implications of environmental diclofenac: role of mineralogy and solar flux. Ecotoxicol Environ Saf 246:114138. 10.1016/j.ecoenv.2022.114138 36201921 10.1016/j.ecoenv.2022.114138

[CR17] Hasan Husien A, Tseberlidis G, Trifiletti V, Fabbretti E, Mostoni S, McGettrick J, Watson T, Po R, Binetti S (2025) Optimized hot injection and HCl purification for high quality Cu_2_ZnSnS_4_ nanoparticles. Nanoscale Adv 7:250–260. 10.1039/d4na00843j 10.1039/d4na00843j PMC11583927 39583134

[CR18] Hemkumar K, Ananthi P, Pius A (2023) Enhanced photocatalytic activity of MIL-88 a impregnated with Ag_3_PO_4_/GCN for the degradation of diclofenac sodium. Mater Sci Eng B 292:116453. 10.1016/j.mseb.2023.116453

[CR19] Henríquez R, Salazar Nogales P, Grez Moreno P, Muñoz Cartagena E, Leyton Bongiorno P, Navarrete-Astorga E, Dalchiele EA (2023) One-step hydrothermal synthesis of Cu2ZnSnS4 nanoparticles as an efficient visible light photocatalyst for the degradation of Congo Red Azo Dye. Nanomaterials 13:1731. 10.3390/nano13111731 37299634 10.3390/nano13111731 PMC10254566

[CR20] Hou X, Li Y, Yan J-J, Wang C-W (2014) Highly efficient photocatalysis of p-type Cu_2_ZnSnS_4_ under visible-light illumination. Mater Res Bull 60:628–633. 10.1016/j.materresbull.2014.09.041

[CR21] ISO 15705:2002 (2002) Water quality - determination of the chemical oxygen demand index (ST-COD) - small-scale sealed-tube method. International Standard Organization for Standardization.

[CR22] Ito K (2014) Copper zinc tin sulfide‐based thin‐film solar cells.Wiley, Pondicherry, India. 10.1002/9781118437865

[CR23] Jiménez-Salcedo M, Monge M, Tena MT (2021) The photocatalytic degradation of sodium diclofenac in different water matrices using g-C_3_N_4_ nanosheets: a study of the intermediate by-products and mechanism. J Environ Chem Eng 9:105827. 10.1016/j.jece.2021.105827

[CR24] Jin X, Wu Y, Zhang Q, Wang F, Chen P, Liu H, Huang S, Wu J, Tu N, Lv W, Liu G (2020) Defect-modified reduced graphitic carbon nitride (RCN) enhanced oxidation performance for photocatalytic degradation of diclofenac. Chemosphere 258:127343. 10.1016/j.chemosphere.2020.127343 32947672 10.1016/j.chemosphere.2020.127343

[CR25] Khanzada LS, Levchuk I, Hou Y, Azimi H, Osvet A, Ahmad R, Brandl M, Herre P, Distaso M, Hock R, Peukert W, Batentschuk M, Brabec CJ (2016) Effective ligand engineering of the Cu2ZnSnS4 nanocrystal surface for increasing hole transport efficiency in perovskite solar cells. Adv Funct Mater 26:8300–8306. 10.1002/adfm.201603441

[CR26] Kodom K, Attiogbe F, Kuranchie FA (2021) Assessment of removal efficiency of pharmaceutical products from wastewater in sewage treatment plants: a case of the sewerage systems Ghana limited. Accra Heliyon 7:e08385. 10.1016/j.heliyon.2021.e08385 34825091 10.1016/j.heliyon.2021.e08385 PMC8605079

[CR27] Kolakovic S, Salgado R, Freitas EB, Bronze MR, Sekulic MT, Carvalho G, Reis MAM, Oehmen A (2022) Diclofenac biotransformation in the enhanced biological phosphorus removal process. Sci Total Environ 806:151232. 10.1016/j.scitotenv.2021.151232 34715209 10.1016/j.scitotenv.2021.151232

[CR28] Kumar A, Škoro N, Gernjak W, Jovanović O, Petrović A, Živković S, Lumbaque EC, Farré MJ, Puač N (2023) Degradation of diclofenac and 4-chlorobenzoic acid in aqueous solution by cold atmospheric plasma source. Sci Total Environ 864:161194. 10.1016/j.scitotenv.2022.161194 36581289 10.1016/j.scitotenv.2022.161194

[CR29] Li X, Zhou M, Pan Y (2018) Degradation of diclofenac by H2O2 activated with pre-magnetization Fe^0^: influencing factors and degradation pathways. Chemosphere 212:853–862. 10.1016/j.chemosphere.2018.08.144 30193234 10.1016/j.chemosphere.2018.08.144

[CR30] Li F, Huang T, Sun F, Chen L, Li P, Shao F, Yang X, Liu W (2022) Ferric oxide nanoclusters with low-spin Fe^III^ anchored g-C_3_N_4_ rod for boosting photocatalytic activity and degradation of diclofenac in water under solar light. Appl Catal B Environ 317:121725. 10.1016/j.apcatb.2022.121725

[CR31] Liu X, Li F, Liu Y, Li P, Chen L, Li B, Qian T, Liu W (2022) Degradation of diclofenac in a photosensitization-like photocatalysis process using palladium quantum dots deposited graphite carbon nitride under solar light. J Environ Chem Eng 10:107545. 10.1016/j.jece.2022.107545

[CR32] Luongo G, Guida M, Siciliano A, Libralato G, Saviano L, Amoresano A, Previtera L, Di Fabio G, Zarrelli A (2021) Oxidation of diclofenac in water by sodium hypochlorite: identification of new degradation by-products and their ecotoxicological evaluation. J Pharm Biomed Anal 194:113762. 10.1016/j.jpba.2020.113762 33248860 10.1016/j.jpba.2020.113762

[CR33] Meroni D, Bianchi CL, Boffito DC, Cerrato G, Bruni A, Sartirana M, Falletta E (2021) Piezo-enhanced photocatalytic diclofenac mineralization over ZnO. Ultrason Sonochem 75:105615. 10.1016/j.ultsonch.2021.105615 34111723 10.1016/j.ultsonch.2021.105615 PMC8193124

[CR34] Murgolo S, Moreira IS, Piccirillo C, Castro PML, Ventrella G, Cocozza C, Mascolo G (2018) Photocatalytic degradation of diclofenac by hydroxyapatite–TiO_2_ composite material: identification of transformation products and assessment of toxicity. Materials 11:1779. 10.3390/ma11091779 30235831 10.3390/ma11091779 PMC6164299

[CR35] Nguyen TP, Tran QB, Ly QV, Thanh Hai L, Le DT, Tran MB, Ho TTT, Nguyen XC, Shokouhimehr M, Vo D-VN, Lam SS, Do H-T, Kim SY, Van Tung T, Van Le Q (2020) Enhanced visible photocatalytic degradation of diclofenac over N-doped TiO_2_ assisted with H_2_O_2_: a kinetic and pathway study. Arab J Chem 13:8361–8371. 10.1016/j.arabjc.2020.05.023

[CR36] Pérez-Estrada LA, Malato S, Gernjak W, Agüera A, Thurman EM, Ferrer I, Fernández-Alba AR (2005) Photo-Fenton degradation of diclofenac: identification of main intermediates and degradation pathway. Environ Sci Technol 39:8300–8306. 10.1021/es050794n 16294867 10.1021/es050794n

[CR37] Phaltane AS, Vanalakar SA, Bhat TS, Patil PS, Sartale SD, Kadam LD (2017) Photocatalytic degradation of by hydrothermally synthesized CZTS nanoparticles. J Mater Sci Mater Electron 28:8186–8191. 10.1007/s10854-017-6527-0

[CR38] Salaeh S, Juretic Perisic D, Biosic M, Kusic H, Babic S, Lavrencic Stangar U, Dionysiou DD, Loncaric Bozic A (2016) Diclofenac removal by simulated solar assisted photocatalysis using TiO_2_-based zeolite catalyst; mechanisms, pathways and environmental aspects. Chem Eng J 304:289–302. 10.1016/j.cej.2016.06.083

[CR39] Salgado R, Pereira VJ, Carvalho G, Soeiro R, Gaffney V, Almeida C, Vale Cardoso V, Ferreira E, Benoliel MJ, Ternes TA, Oehmen A, Reis MAM, Noronha JP (2013) Photodegradation kinetics and transformation products of ketoprofen, diclofenac and atenolol in pure water and treated wastewater. J Hazard Mater 244–245:516–527. 10.1016/j.jhazmat.2012.10.039 10.1016/j.jhazmat.2012.10.039 23177274

[CR40] Sarasidis VC, Plakas KV, Patsios SI, Karabelas AJ (2014) Investigation of diclofenac degradation in a continuous photo-catalytic membrane reactor. Influence of operating parameters. Chem Eng J 239:299–311. 10.1016/j.cej.2013.11.026

[CR41] Semalti P, Sharma V, Sharma SN (2021) A novel method of water remediation of organic pollutants and industrial wastes by solution- route processed CZTS nanocrystals. J Materiomics 7:904–919. 10.1016/j.jmat.2021.04.005

[CR42] Silvestri S, Ferreira CD, Oliveira VJ, Varejão MTB, Labrincha JA, Tobaldi DM (2019) Synthesis of PPy-ZnO composite used as photocatalyst for the degradation of diclofenac under simulated solar irradiation. J Photochem Photobiol A Chem 375:261–269. 10.1016/j.jphotochem.2019.02.034

[CR43] Sobhani K, Li J, Cortes M (2023) Nonsteroidal anti-inflammatory drugs (NSAIDs). StatPearls Publishing10.1007/978-3-031-21291-8_8

[CR44] Ternes T, Joss A (2006) Human pharmaceuticals, hormones and fragrances - the challenge of micropollutants in urban water management. IWA Pub, London

[CR45] TEST (2024) Ver. 5.1.2. Available online: https://www.epa.gov/chemical-research/toxicity-estimation-software-tool-test. Accessed 2 May 2025

[CR46] Trifiletti V, Mostoni S, Butrichi F, Acciarri M, Binetti S, Scotti R (2019) Study of precursor-inks designed for high-quality Cu_2_ZnSnS_4_ films for low-cost PV application. ChemistrySelect 4:4905–4912. 10.1002/slct.201900170

[CR47] Tseberlidis G, Trifiletti V, Le Donne A, Frioni L, Acciarri M, Binetti S (2020) Kesterite solar-cells by drop-casting of inorganic sol–gel inks. Sol Energy 208:532–538. 10.1016/j.solener.2020.07.093

[CR48] Tseberlidis G, Hasan Husien A, Riva S, Frioni L, Le Donne A, Acciarri M, Binetti S (2021) Semi-transparent Cu_2_ZnSnS_4_ solar cells by drop-casting of sol-gel ink. Sol Energy 224:134–141. 10.1016/j.solener.2021.05.073

[CR49] Tseberlidis G, Trifiletti V, Hasan Husien A, L’Altella A, Binetti S, Gosetti F (2024) Cu_2_ZnSnS_4_ nanoparticles as an efficient photocatalyst for the degradation of diclofenac in water. Appl Sci 14:9923. 10.3390/app14219923

[CR50] Tsugawa H, Cajka T, Kind T, Ma Y, Higgins B, Ikeda K, Kanazawa M, VanderGheynst J, Fiehn O, Arita M (2015) MS-DIAL: data-independent MS/MS deconvolution for comprehensive metabolome analysis. Nat Methods 12:523–526. 10.1038/nmeth.3393 25938372 10.1038/nmeth.3393 PMC4449330

[CR51] Tsugawa H, Kind T, Nakabayashi R, Yukihira D, Tanaka W, Cajka T, Saito K, Fiehn O, Arita M (2016) Hydrogen rearrangement rules: computational MS/MS fragmentation and structure elucidation using MS-FINDER software. Anal Chem 88:7946–7958. 10.1021/acs.analchem.6b00770 27419259 10.1021/acs.analchem.6b00770 PMC7063832

[CR52] U.S. Environmental Protection Agency (2020) User’s guide for T. E. S. T. (Toxicity Estimation Software Tool) version 5.1.63. https://www.epa.gov/chemical-research/toxicity-estimation-software-tool-test. Accessed 2 May 2025

[CR53] United Nations (2021) Globally harmonized system of classification and labelling of chemicals (GHS), ninth revised ed., New York and Geneva. https://unece.org/sites/default/files/2021-09/GHS_Rev9E_0.pdf. Accessed 2 May 2025

[CR54] VEGA QSAR (2024) Ver. 1.2.0. Available online: https://www.vegahub.eu/portfolio-item/vega-qsar/. Accessed 2 May 2025

[CR55] Wang Y, Song Z, Zhang L, Dong D, Li Z, Sun H, Wang L, Guo Z (2023) Distribution and photodegradation of typical nonsteroidal anti-inflammatory drugs in an ice-water system: simulation of surface waters with an ice cover. J Clean Prod 402:136823. 10.1016/j.jclepro.2023.136823

[CR56] Wei Q-B, Xu P, Ren X-P, Fu F (2019) Flower-like Cu2ZnSnS4 architectures synthetize and their visible-light catalytic properties. J Alloys Compd 770:424–432. 10.1016/j.jallcom.2018.08.140

[CR57] Wu MH, Shi J, Deng HP (2018) Metal doped manganese oxide octahedral molecular sieve catalysts for degradation of diclofenac in the presence of peroxymonosulfate. Arab J Chem 11:924–934. 10.1016/j.arabjc.2018.02.002

[CR58] Yang Y, Ok YS, Kim K-H, Kwon EE, Tsang YF (2017) Occurrences and removal of pharmaceuticals and personal care products (PPCPs) in drinking water and water/sewage treatment plants: a review. Sci Total Environ 596–597:303–320. 10.1016/j.scitotenv.2017.04.102 10.1016/j.scitotenv.2017.04.102 28437649

[CR59] Zaki MF, Wu Y-H, Chen P-C, Chen P-S (2023) Determination of psychoactive substances in one microliter plasma using a novel 3D printing microfluidic paper-based column coupled to liquid chromatography-mass spectrometry. Sens Actuat B Chem 393:134243. 10.1016/j.snb.2023.134243

[CR60] Zhang Y, Geißen S-U, Gal C (2008) Carbamazepine and diclofenac: removal in wastewater treatment plants and occurrence in water bodies. Chemosphere 73:1151–1161. 10.1016/j.chemosphere.2008.07.086 18793791 10.1016/j.chemosphere.2008.07.086

[CR61] Zhou T, Feng K, Xiang W, Lv Y, Wu X, Mao J, He C (2018) Rapid decomposition of diclofenac in a magnetic field enhanced zero-valent iron/EDTA Fenton-like system. Chemosphere 193:968–977. 10.1016/j.chemosphere.2017.11.090 29874773 10.1016/j.chemosphere.2017.11.090

[CR62] Zhu X, Liu S, Gao X, Gu Y, Yu Y, Li M, Chen X, Fan M, Jia Y, Tian L, Xiang M, Yu Y (2024) Typical emerging contaminants in sewage treatment plant effluent, and related watersheds in the Pearl River Basin: ecological risks and source identification. J Hazard Mater 476:135046. 10.1016/j.jhazmat.2024.135046 38964038 10.1016/j.jhazmat.2024.135046

