# Supplementary material for: Identification and ecotoxicity of the diclofenac transformation products formed by photolytic and photocatalytic processes
Source: Environ Sci Pollut Res Int. 2025 May 5;32(21):12700–12. doi: 10.1007/s11356-025-36466-5 (PMC12119384; doi:10.1007/s11356-025-36466-5)
Supplement: Supplementary file 1 — Supplementary file1 (PDF 3616 KB) [file 11356_2025_36466_MOESM1_ESM.pdf]

SUPPLEMENTARY MATERIALS

IDENTIFICATION AND ECOTOXICITY OF THE DICLOFENAC TRANSFORMATION PRODUCTS FORMED BY PHOTOLYTIC AND PHOTOCATALYTIC PROCESSES

Enmanuel Cruz Muñoz [a], Giorgio Tseberlidis [b,c], Amin Hasan Husien [b], Simona Binetti [b], Fabio Gosetti [a, d,*]

[a]*Department of Earth and Environmental Sciences - DISAT, University of Milano-Bicocca, Piazza della Scienza 1, 20126, Milano, Italy*
[b]*Department of Materials Science and Solar Energy Research Center (MIB-SOLAR), University of Milano-Bicocca, Via Cozzi 55, 20125, Milano, Italy*
[c]*CNR-ISSMC Istituto di Scienza, Tecnologia e Sostenibilità per lo sviluppo dei Materiali Ceramici, Via Granarolo, 64, 48018 Faenza (RA), Italy*
[d]*POLARIS Research Center, University of Milano-Bicocca, Piazza della Scienza 1, 20126, Milano, Italy*

*Corresponding author: Fabio Gosetti, fabio.gosetti@unimib.it

Index

Figure S1. CZTS nanoparticles images acquired with SEM (a) and TEM (b).
Figure S2. Extracted ion chromatogram for DCF (black trace) and TP1, TP2, TP3, TP4, and TP5 (color traces) formed in the UV-Vis photolysis experiments. The chromatographic peak of each TP was selected at the degradation time of its maximum intensity. All the peak intensities are min-max scaled.
Figure S3. Evolution profile of DCF and the TPs identified in UV-Vis photolysis experiments.
Figure S4. Evolution profile of DCF and TPs identified in the UV-Vis photocatalysis experiments.
Figure S5. Extracted ion chromatogram for DCF (black trace) and TPs (color traces) formed in the Vis photocatalysis experiments. The chromatographic peak of each TP was selected at the degradation time of its maximum intensity. All the peak intensities are min-max scaled.
Figure S6. Evolution profile of DCF and TPs identified in the Vis photocatalysis experiments.
Figure S7. MS/MS spectrum of DCF (ESI+).
Figure S8. MS/MS spectrum of DCF (ESI-).
Figure S9. MS/MS spectrum of TP1 (ESI+).
Figure S10. MS/MS spectrum of TP2 (ESI+).
Figure S11. MS/MS spectrum of TP2 (ESI-).
Figure S12. MS/MS spectrum of TP3 (ESI-).
Figure S13. MS/MS spectrum of TP4 ESI+).
Figure S14. MS/MS spectrum of TP5 (ESI-).
Figure S15. MS/MS spectrum of TP6 (ESI-).
Figure S16. MS/MS spectrum of TP7 ESI+).
Figure S17. MS/MS spectrum of TP8 (ESI-).
Figure S18. MS/MS spectrum of TP9 (ESI-).
Figure S19. MS/MS spectrum of TP10 (ESI-).
Figure S20. MS/MS spectrum of TP11 (ESI-).
Figure S21. MS/MS spectrum of TP12 ESI+).
Figure S22. MS/MS spectrum of TP12 (ESI-).

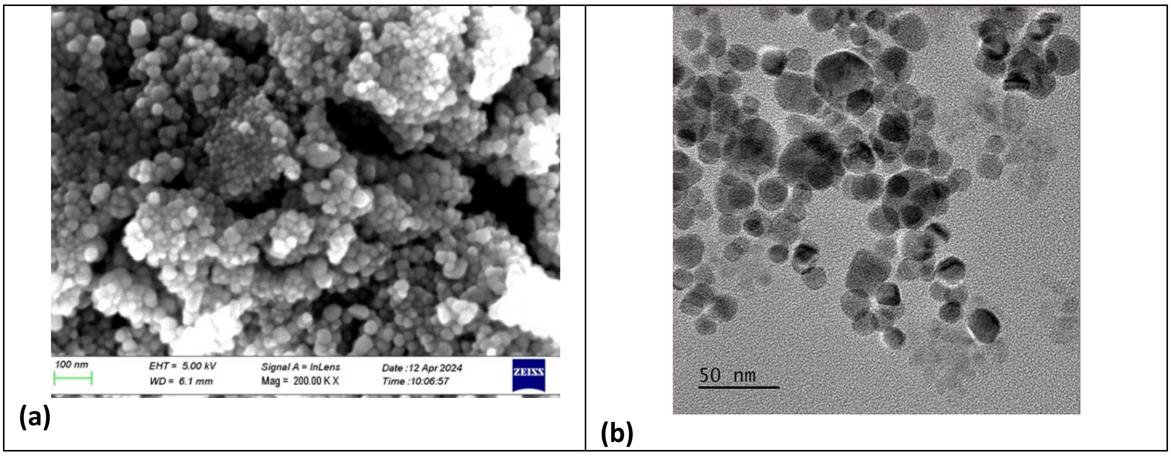

Figure S1. CZTS nanoparticles images acquired with SEM (a) and TEM (b).

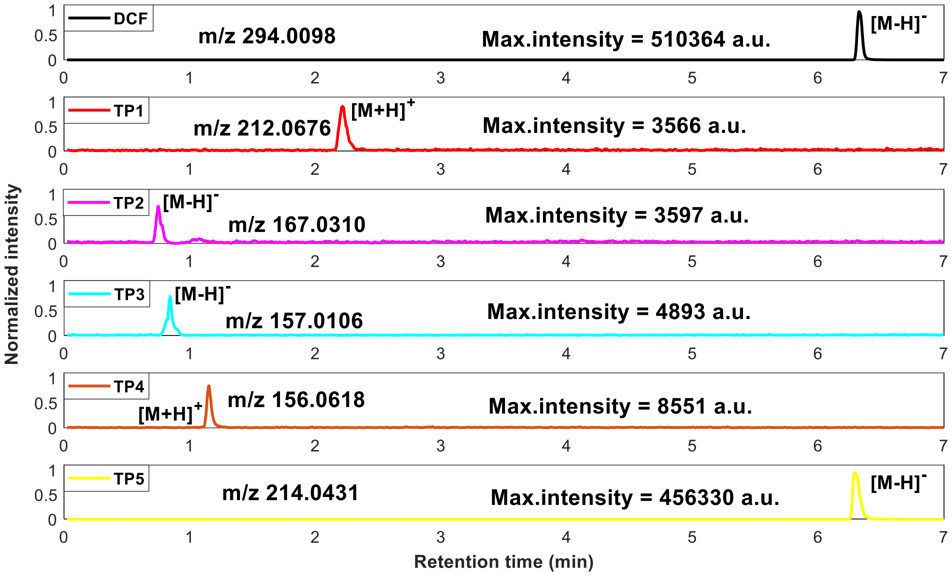

Figure S2. Extracted ion chromatogram for DCF (black trace) and TP1, TP2, TP3, TP4, and TP5 (color traces) formed in the UV-Vis photolysis experiments. The chromatographic peak of each TP was selected at the degradation time of its maximum intensity. All the peak intensities are min-max scaled.

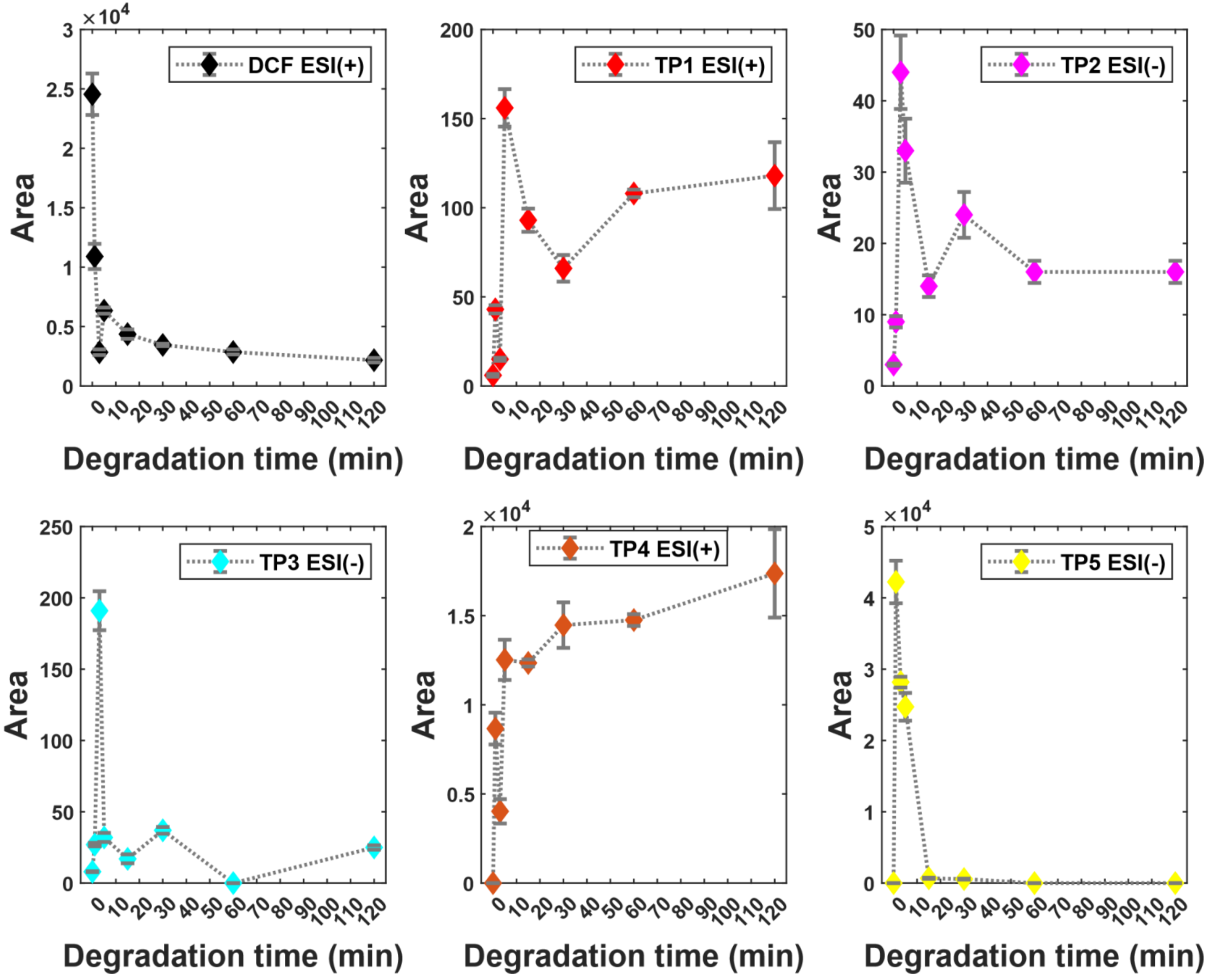

Figure S3. Evolution profile of DCF and the TPs identified in UV-Vis photolysis experiments.

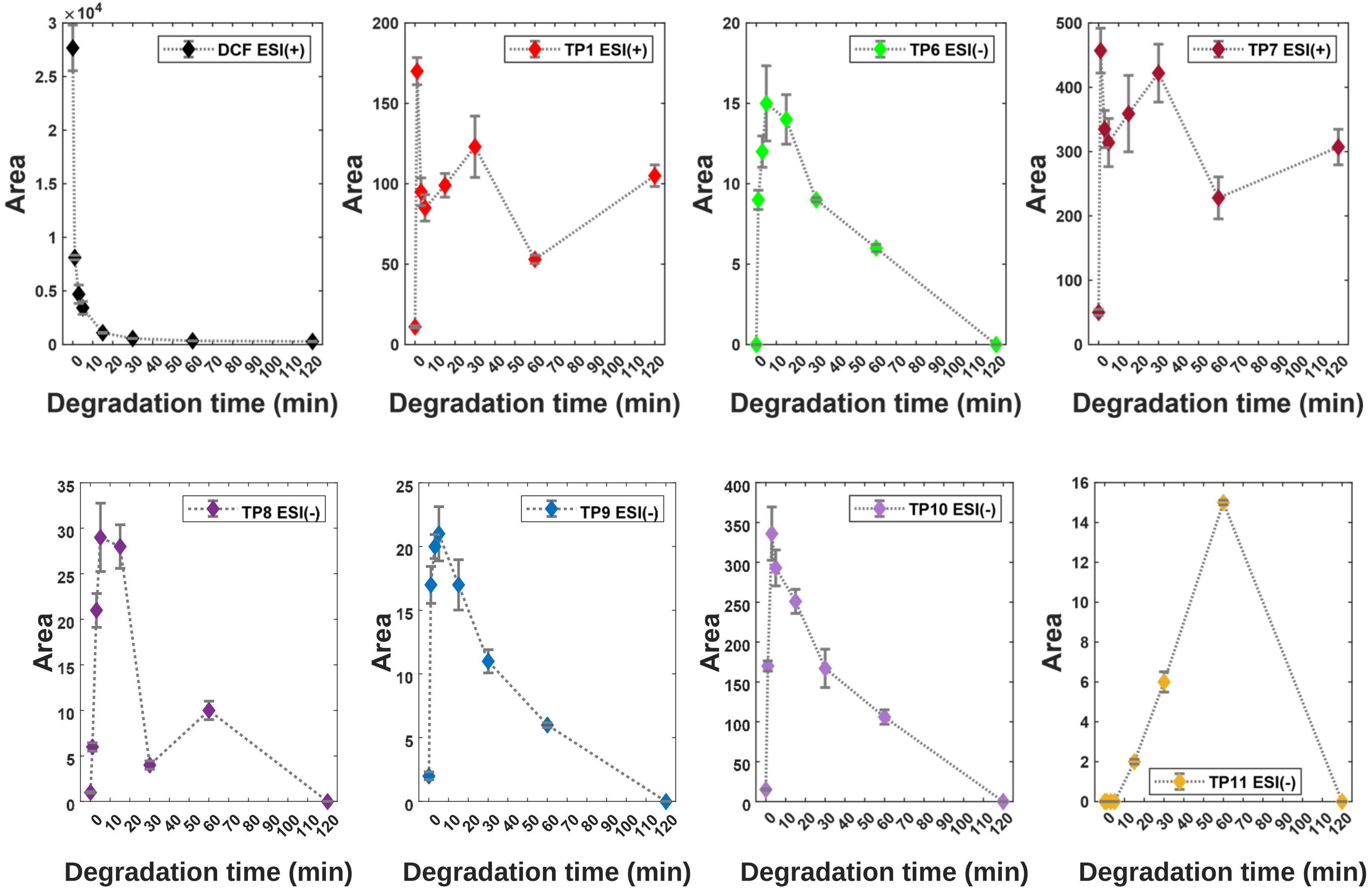

Figure S4. Evolution profile of DCF and TPs identified in the UV-Vis photocatalysis experiments.

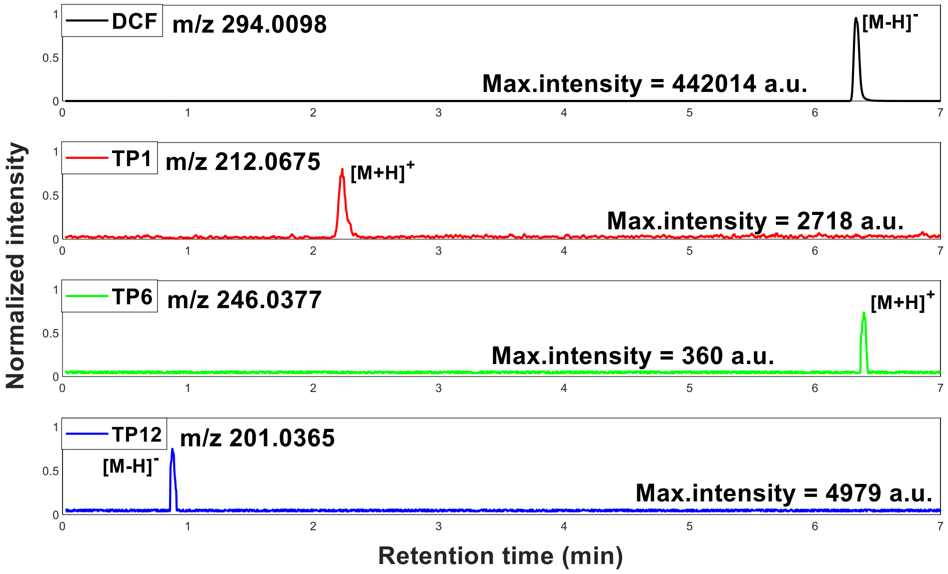

Figure S5. Extracted ion chromatogram for DCF (black trace) and TPs (color traces) formed in the Vis photocatalysis experiments. The chromatographic peak of each TP was selected at the degradation time of its maximum intensity. All the peak intensities are min-max scaled.

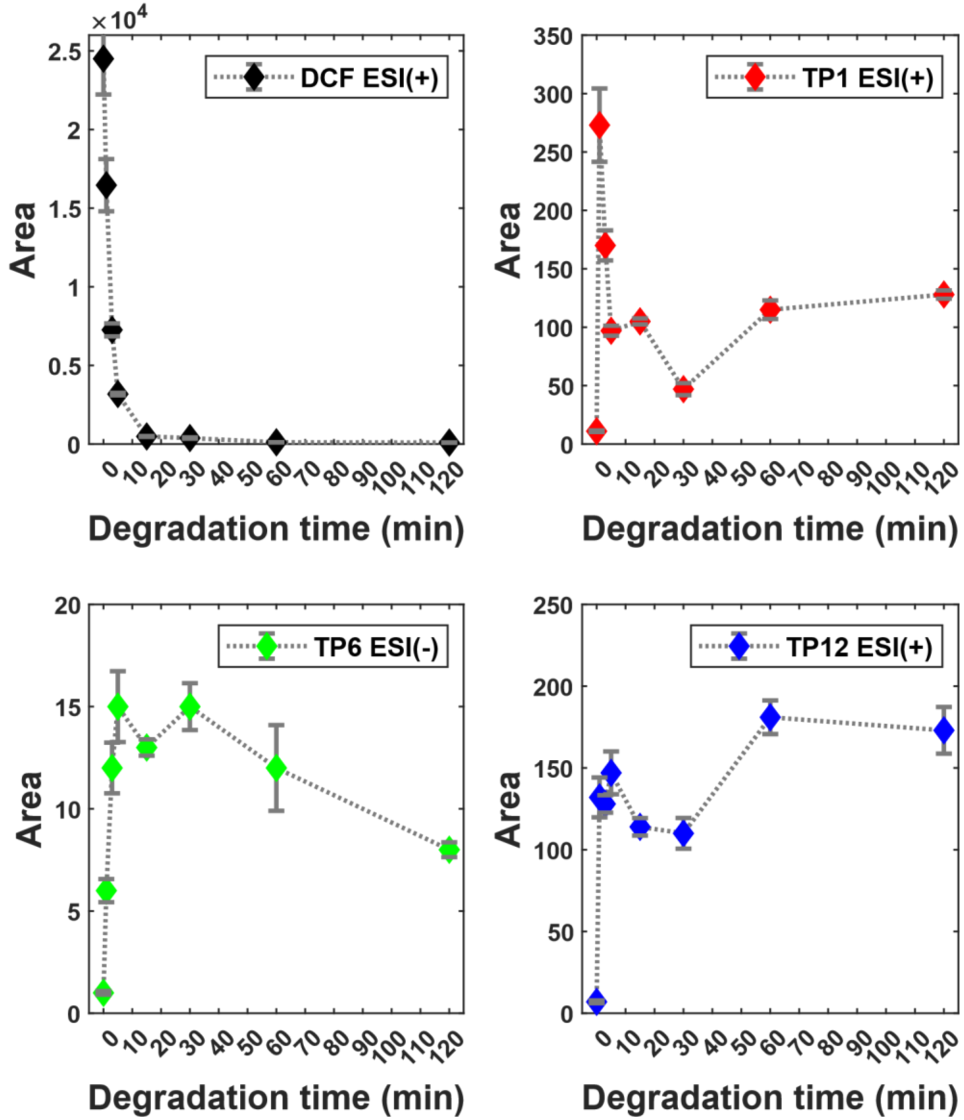

Figure S6. Evolution profile of DCF and TPs identified in the Vis photocatalysis experiments.

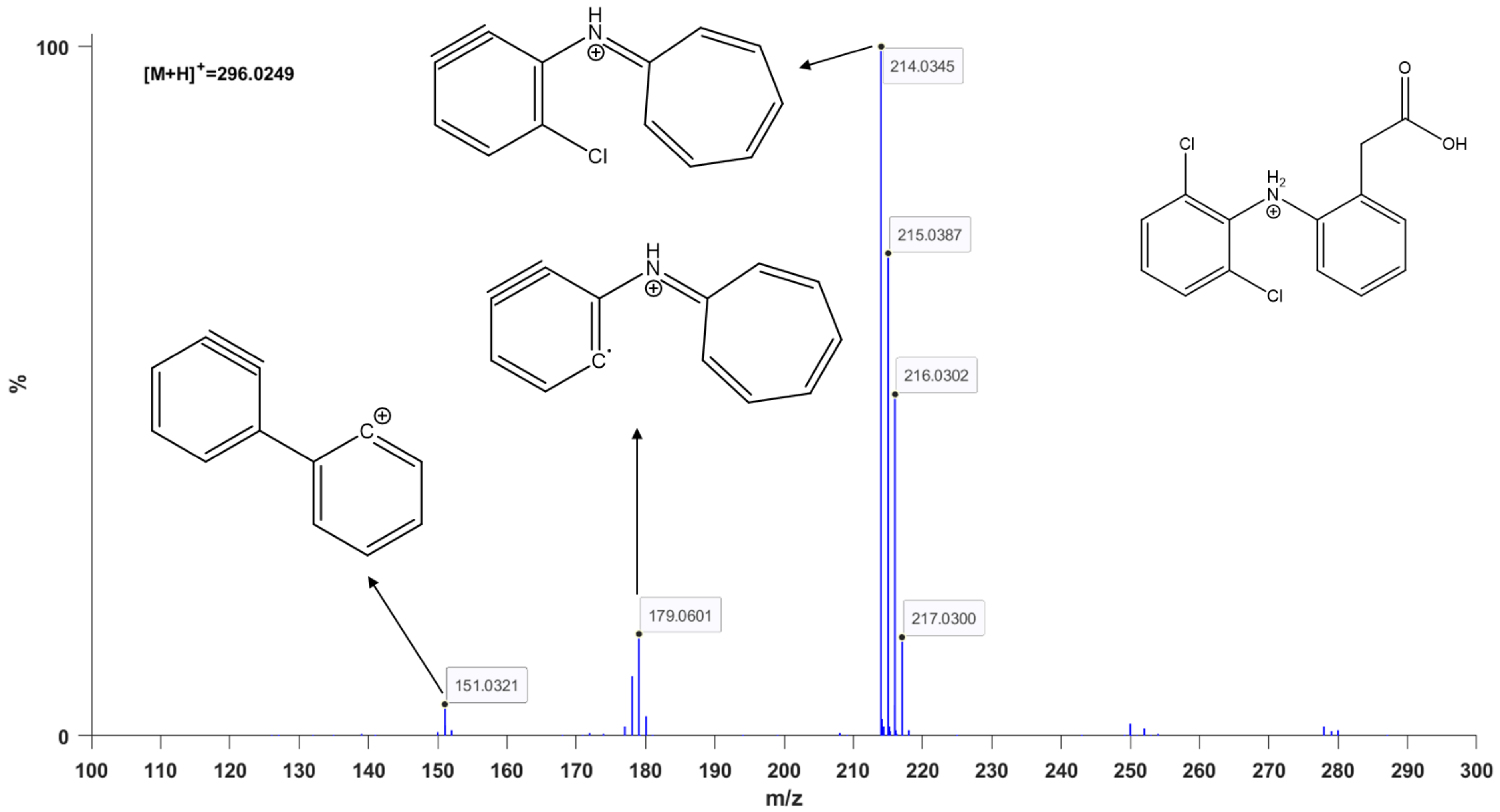

Figure S7. MS/MS spectrum of DCF (ESI+).

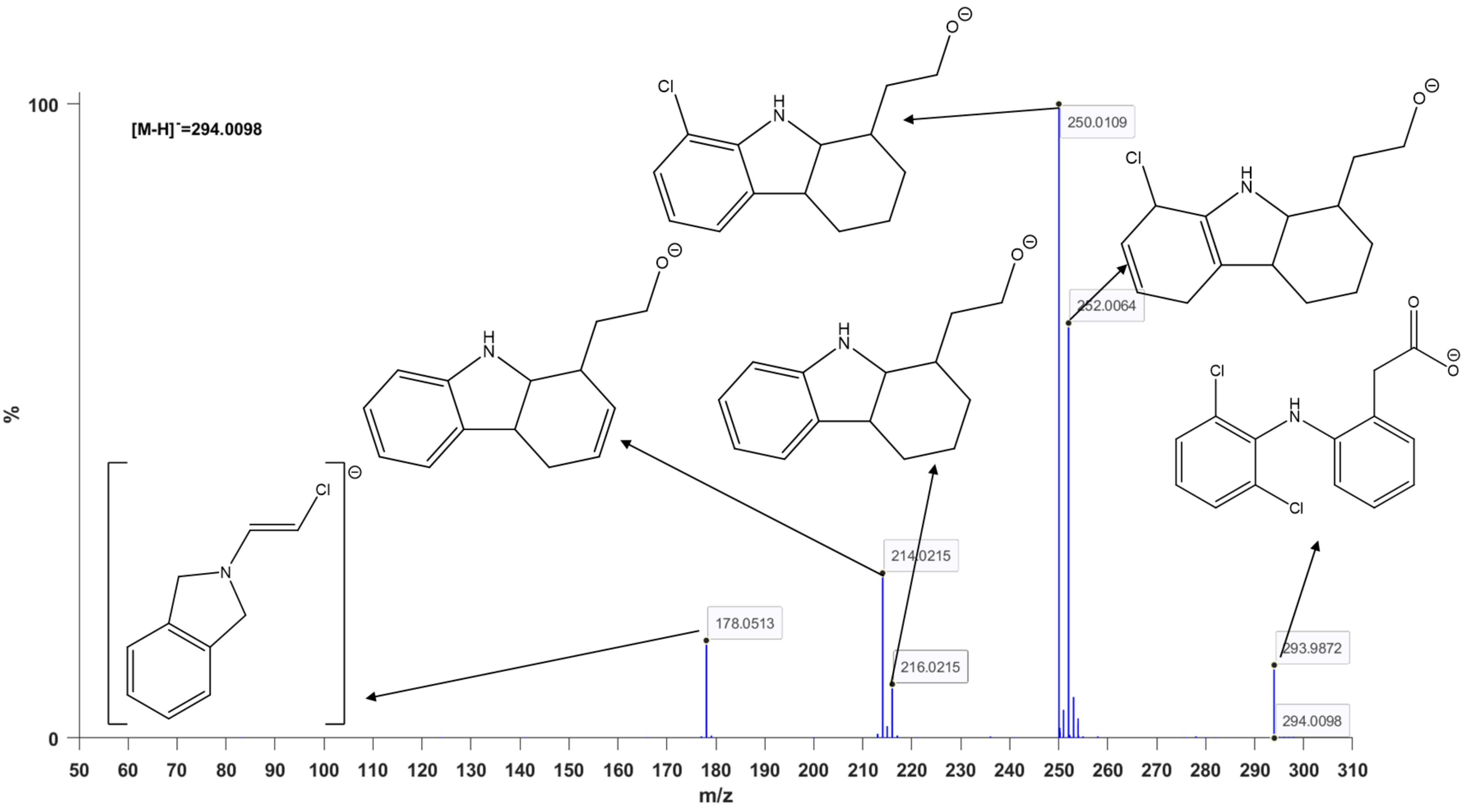

Figure S8. MS/MS spectrum of DCF (ESI-).

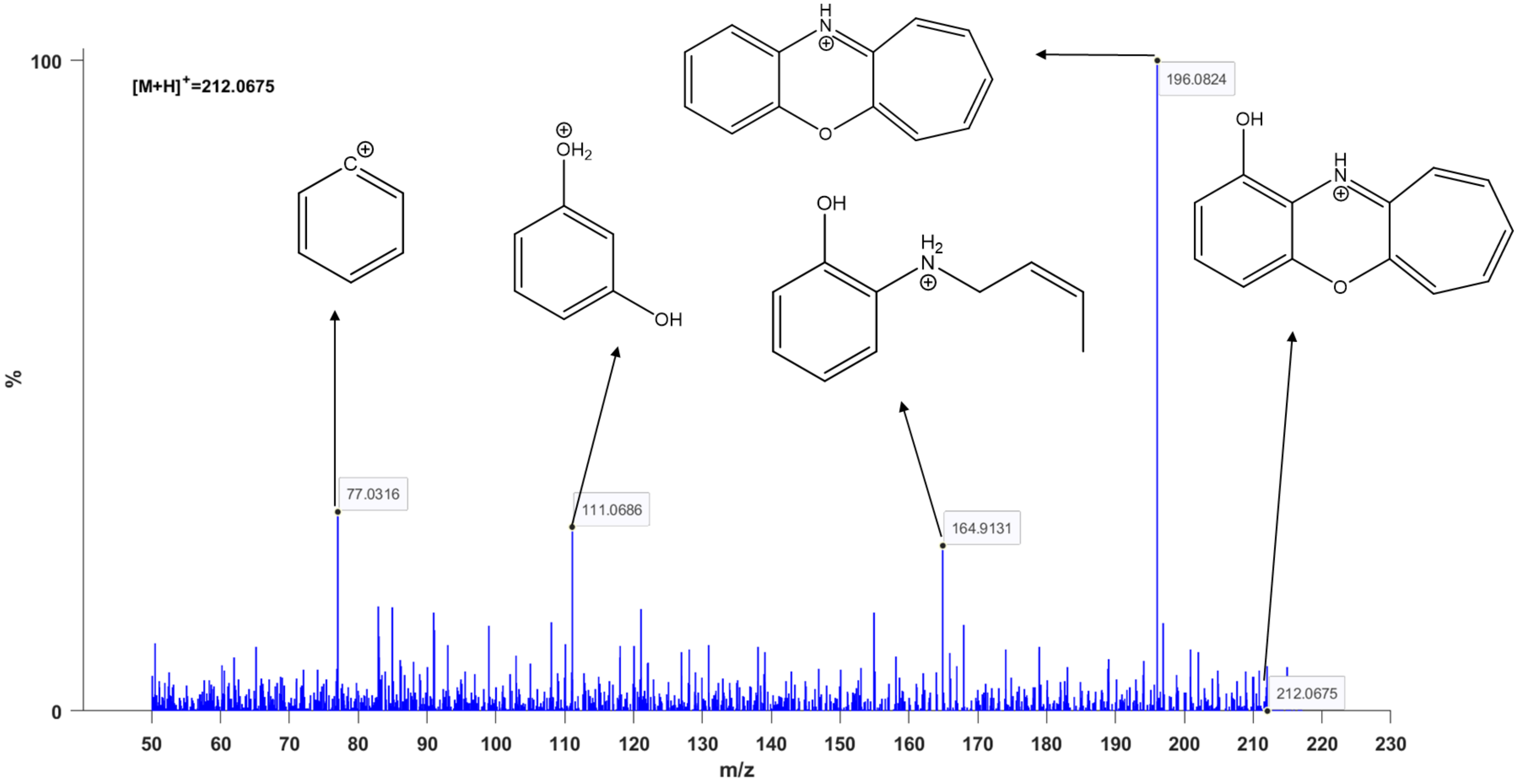

Figure S9. MS/MS spectrum of TP1 (ESI+).

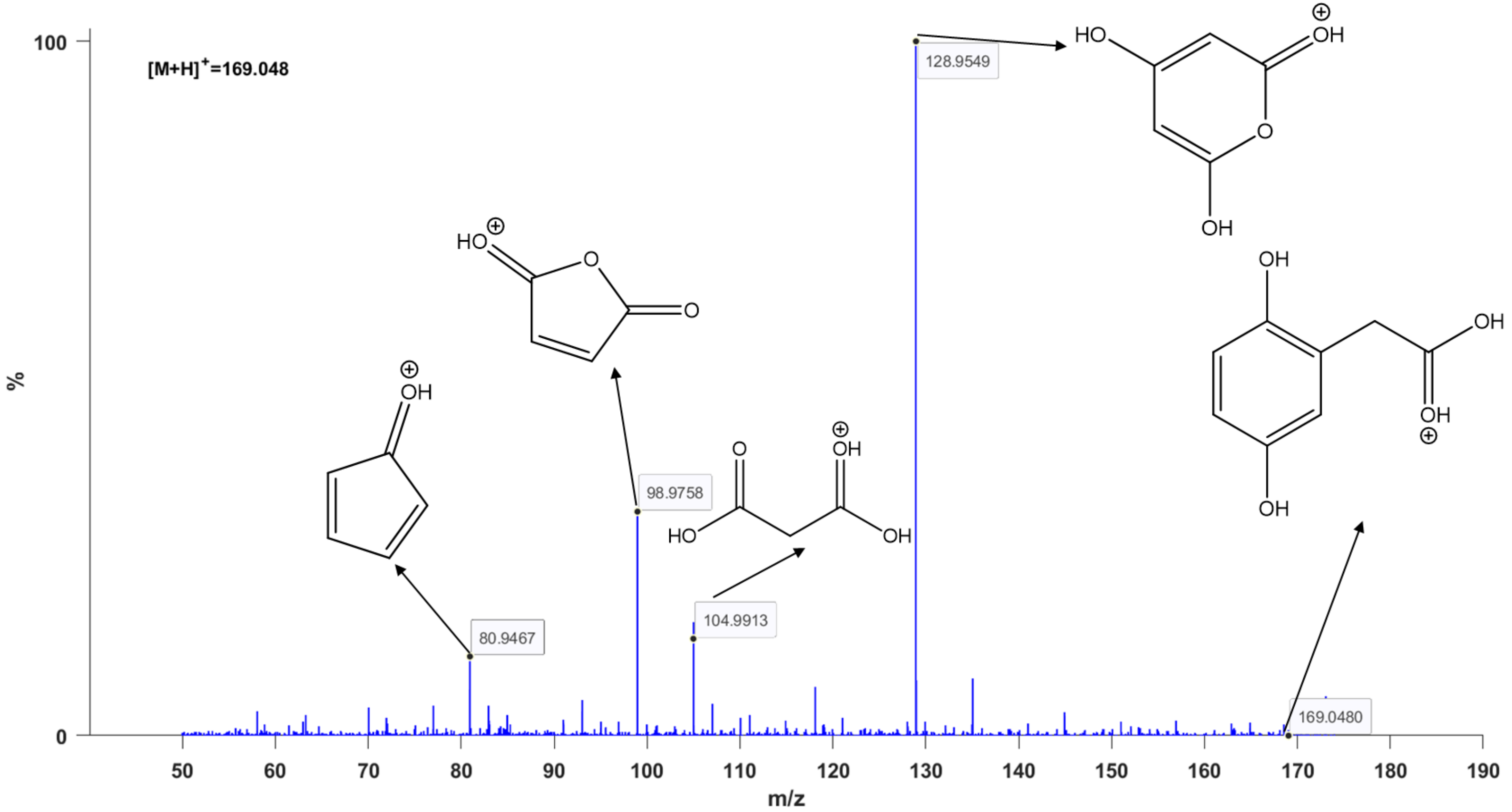

Figure S10. MS/MS spectrum of TP2 (ESI+).

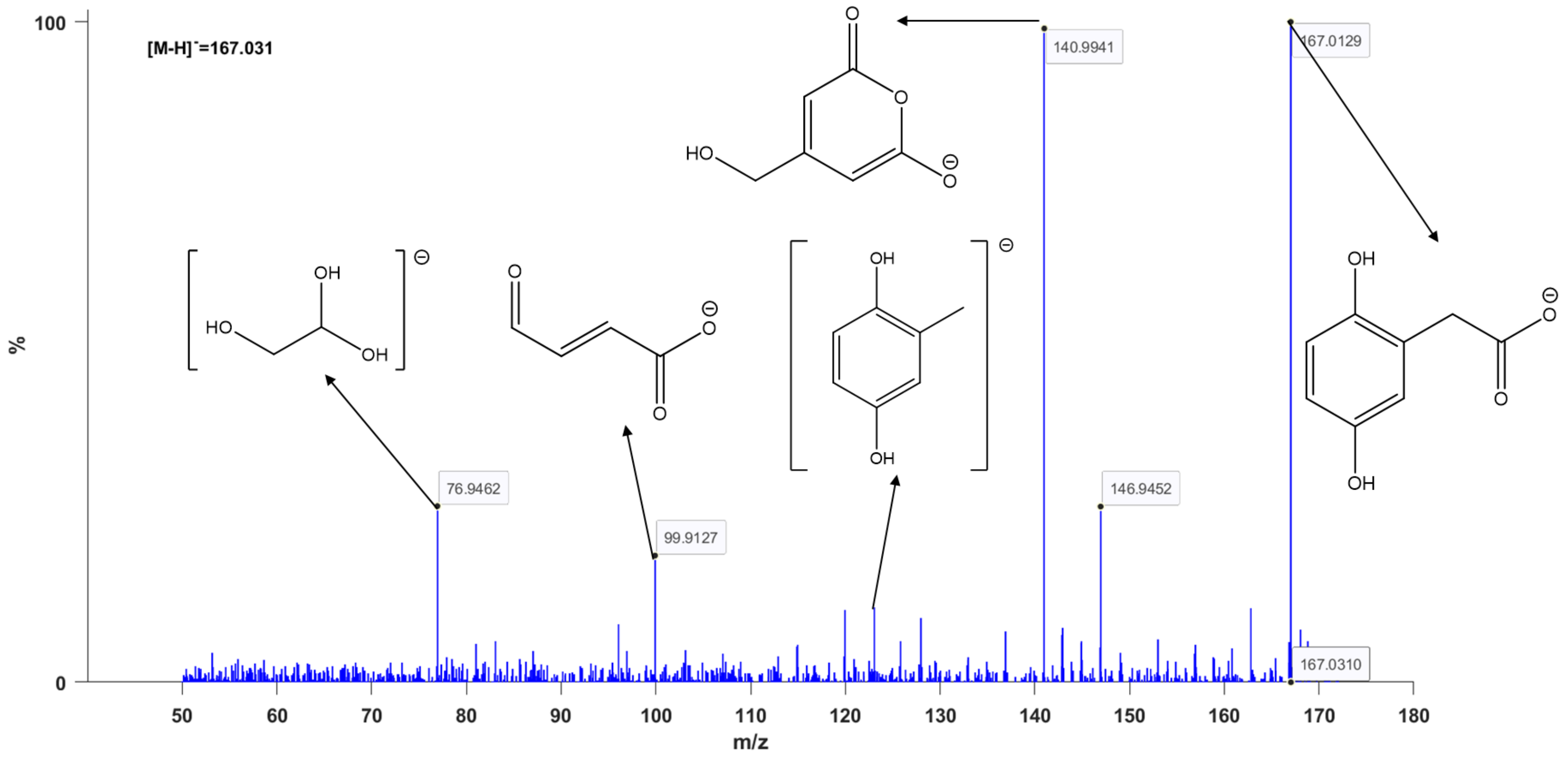

Figure S11. MS/MS spectrum of TP2 (ESI-).

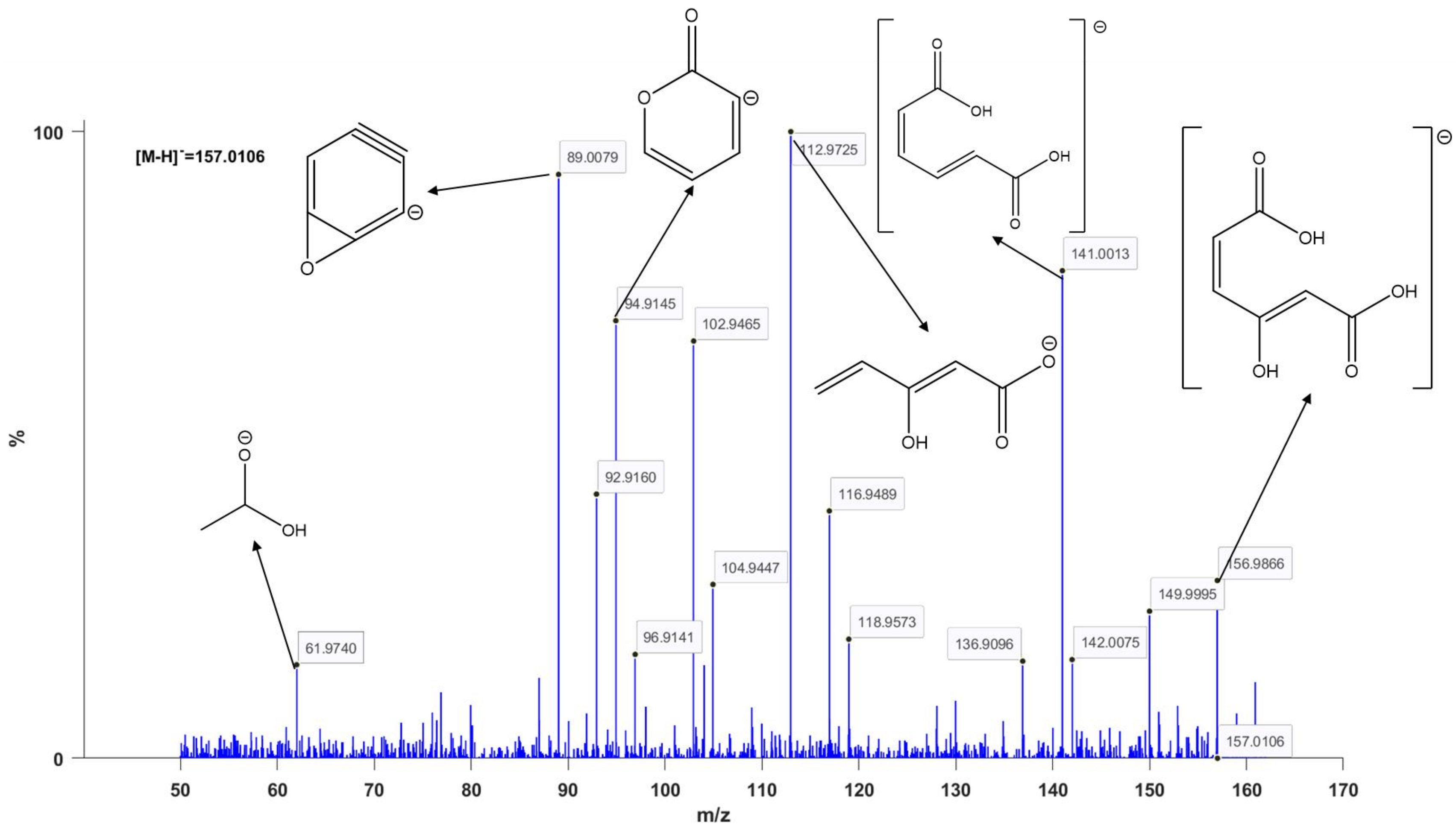

Figure S12. MS/MS spectrum of TP3 (ESI-).

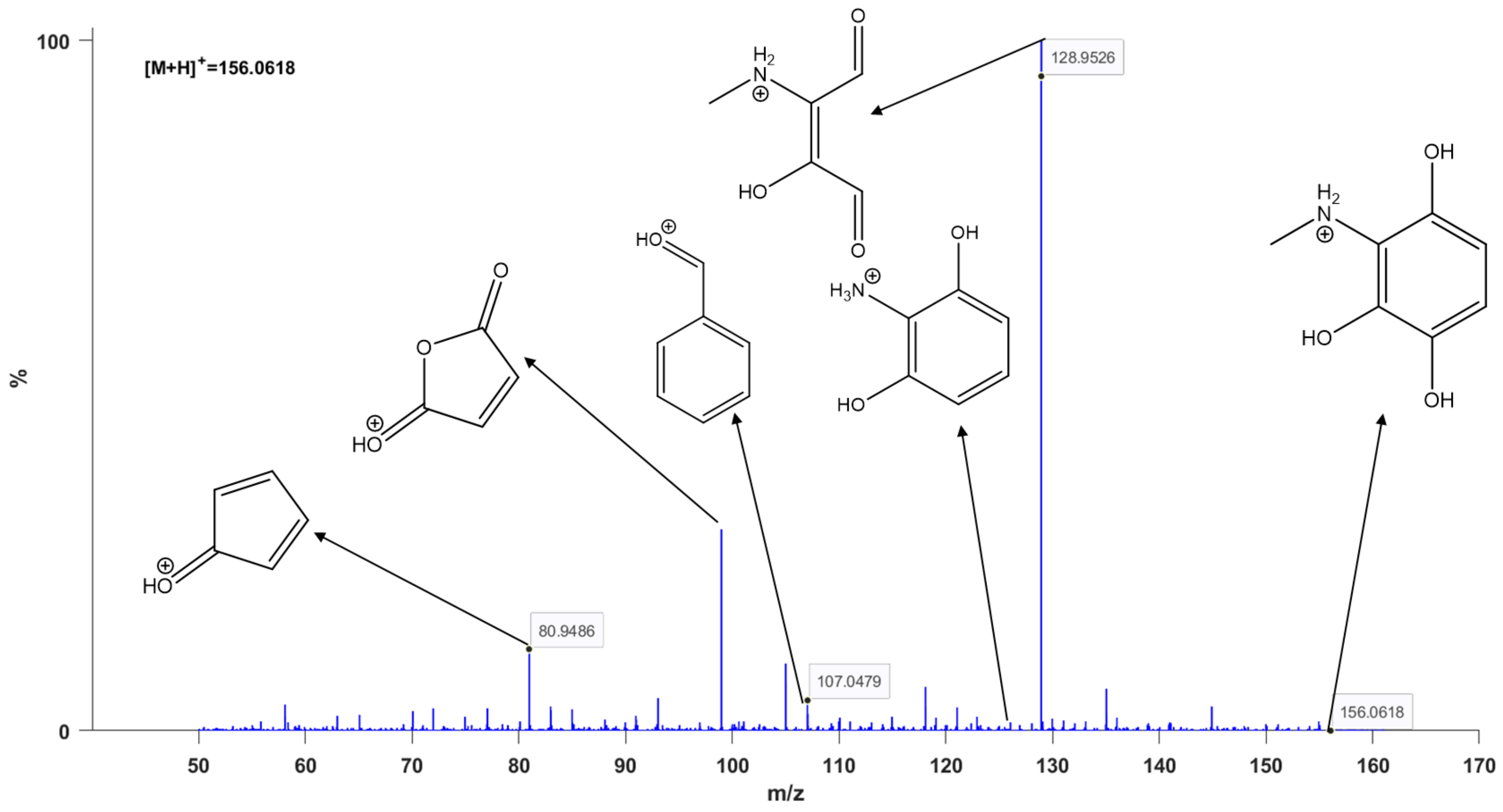

Figure S13. MS/MS spectrum of TP4 ESI+).

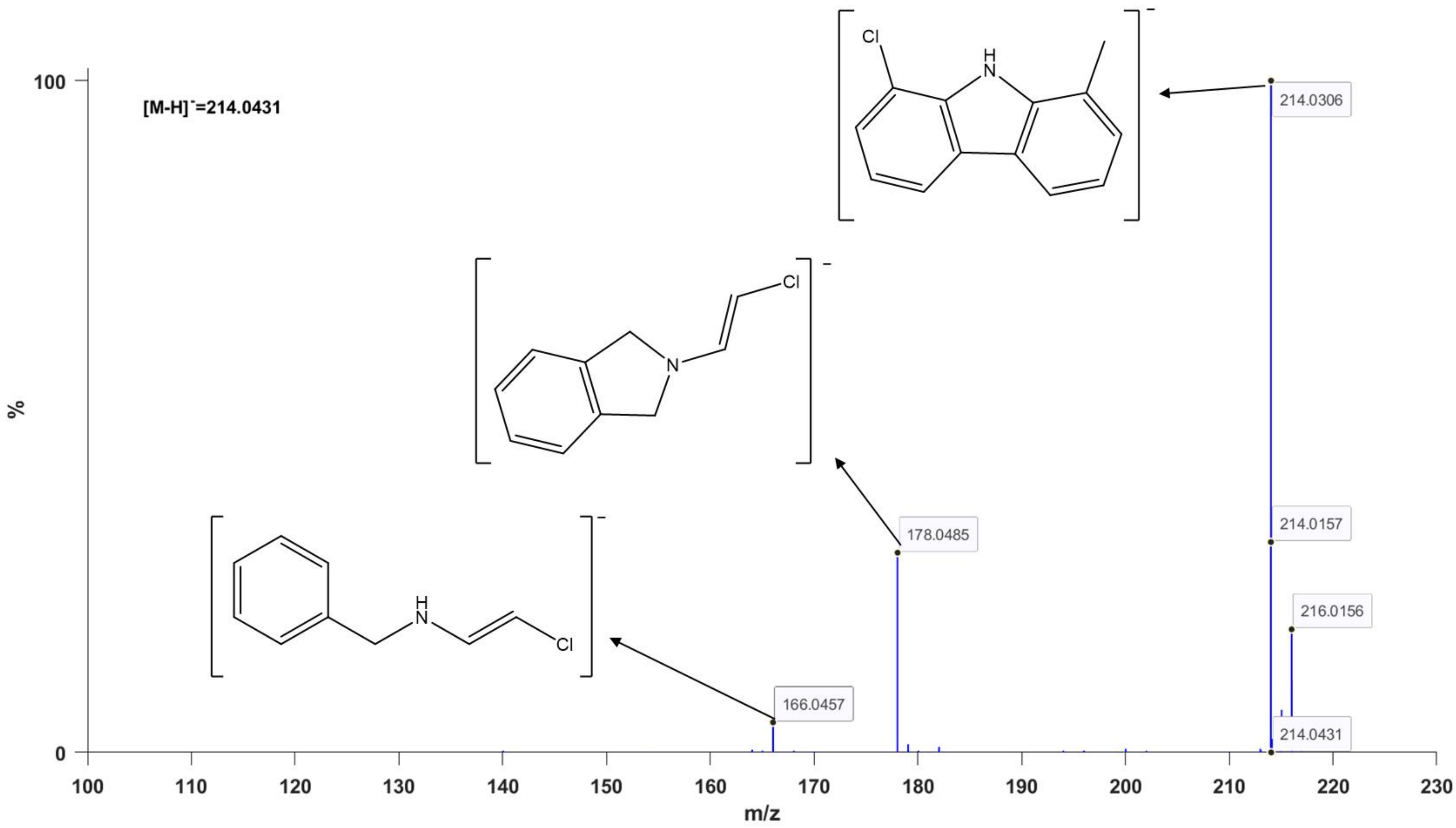

Figure S14. MS/MS spectrum of TP5 (ESI-).

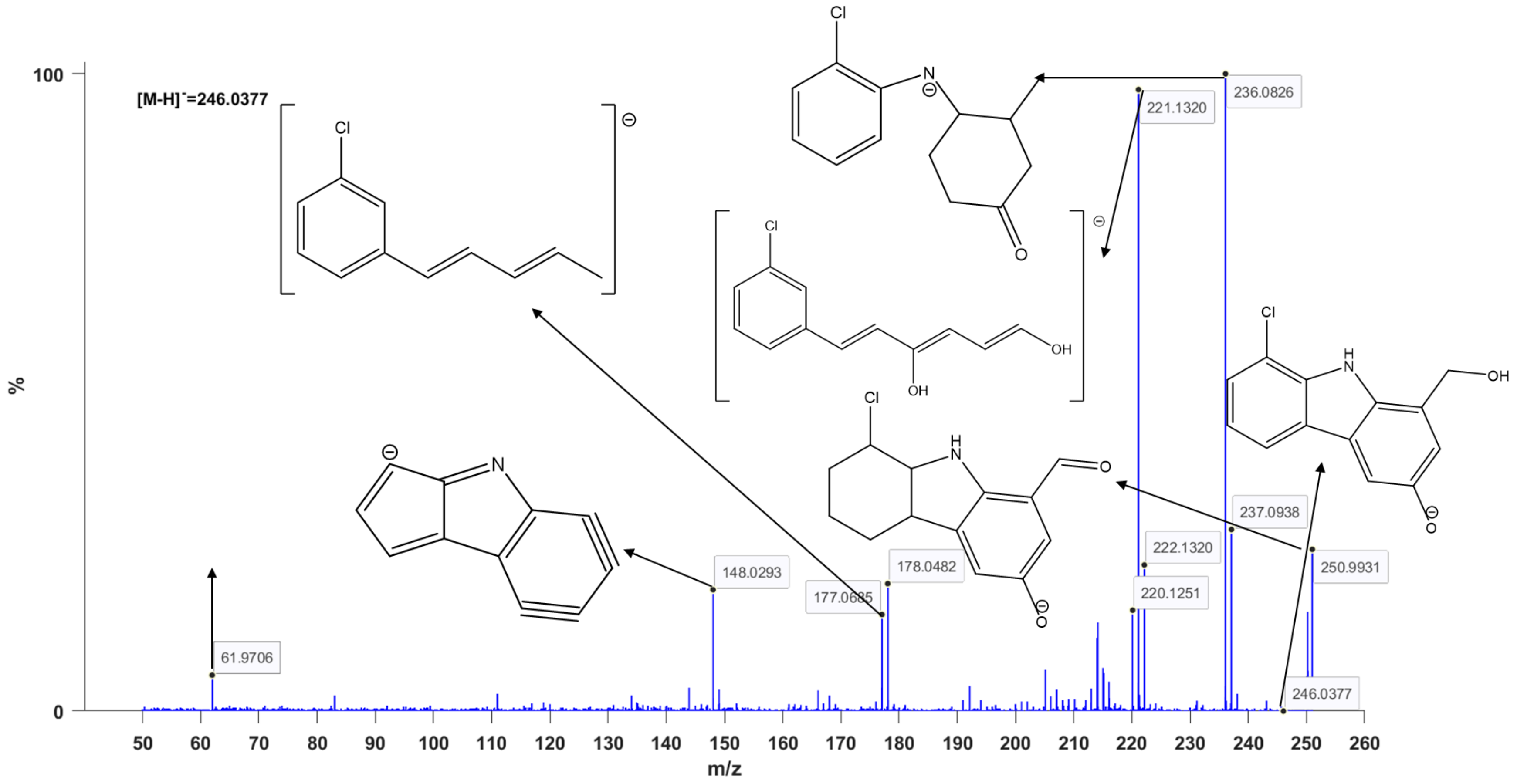

Figure S15. MS/MS spectrum of TP6 (ESI-).

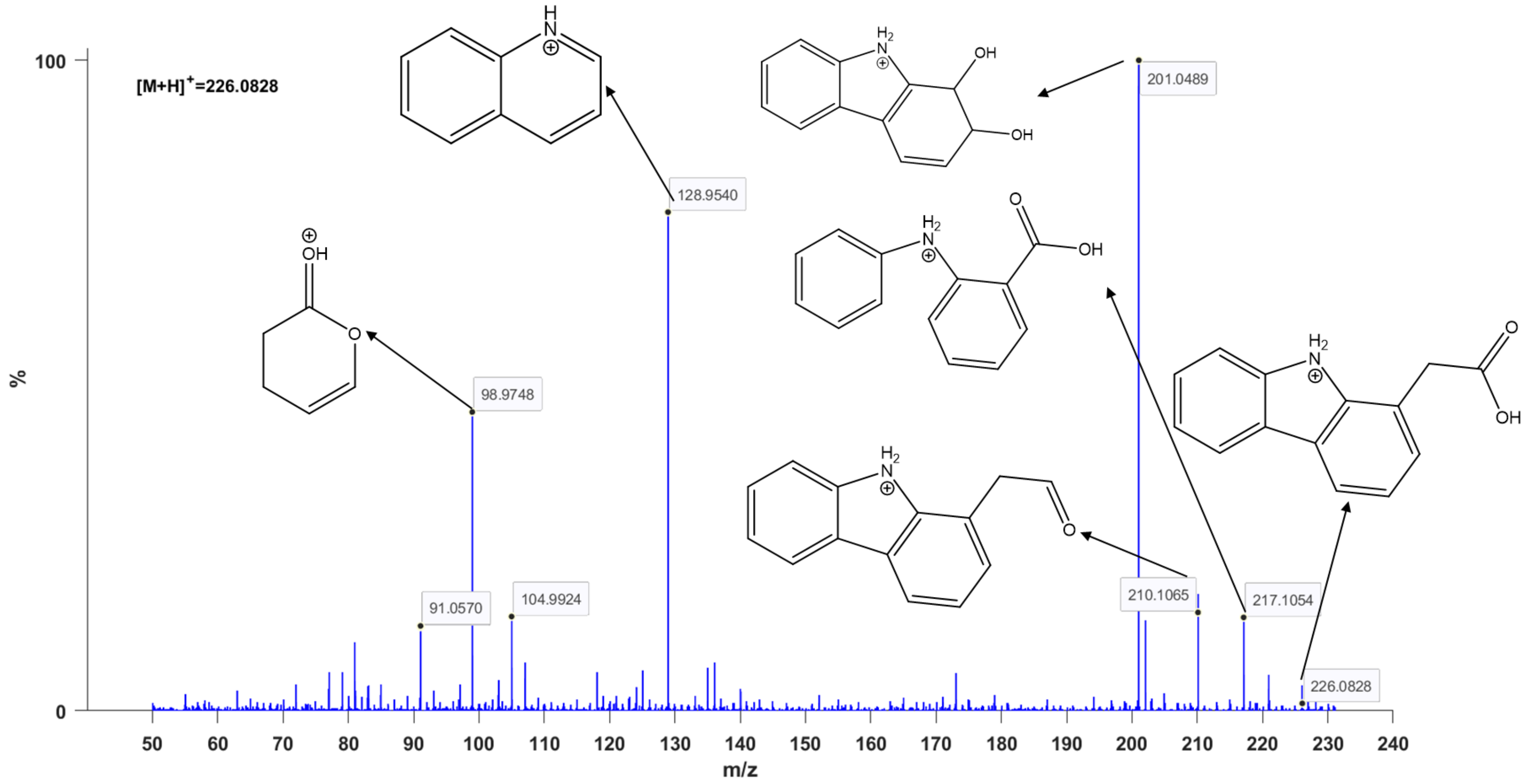

Figure S16. MS/MS spectrum of TP7 ESI+).

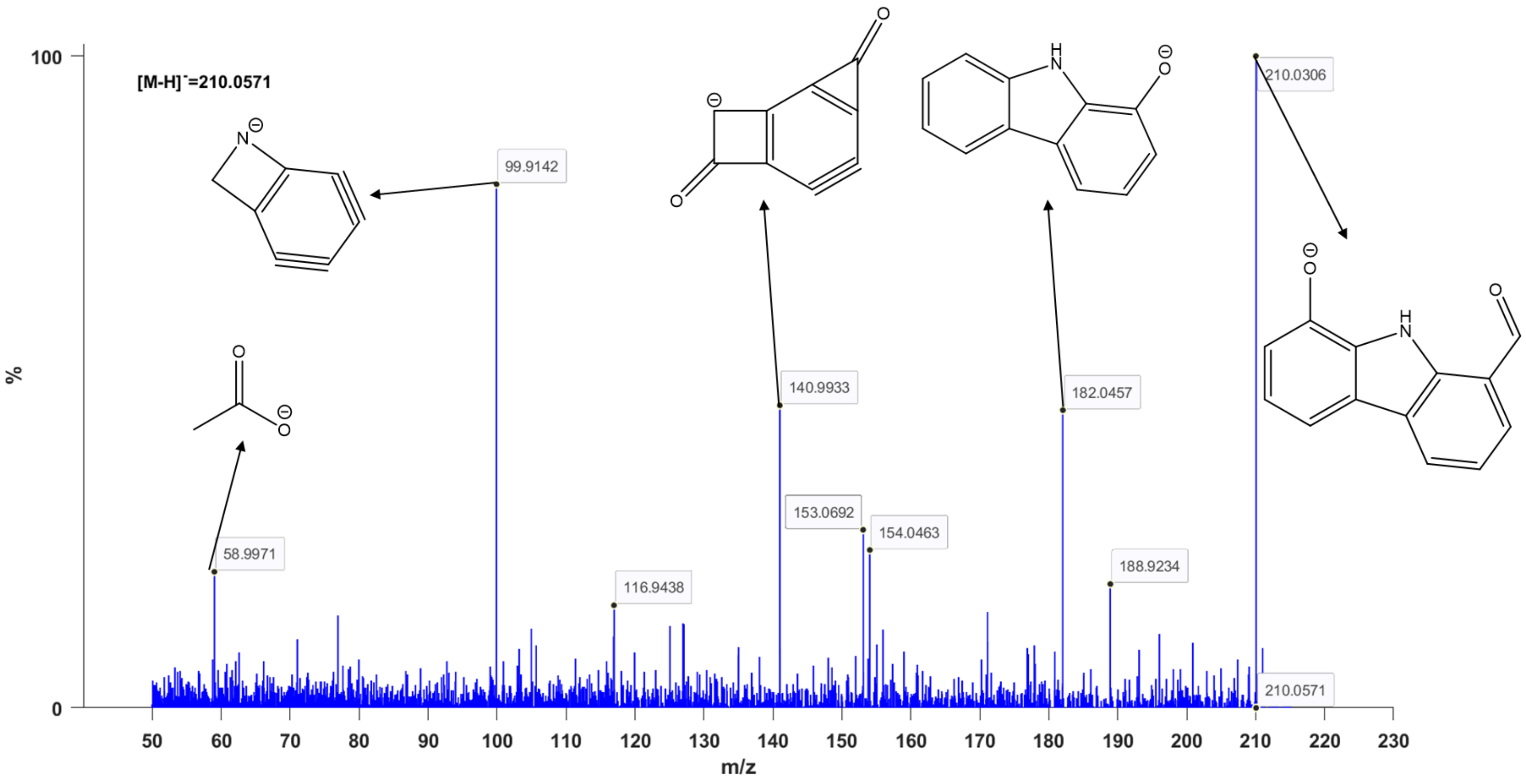

Figure S17. MS/MS spectrum of TP8 (ESI-).

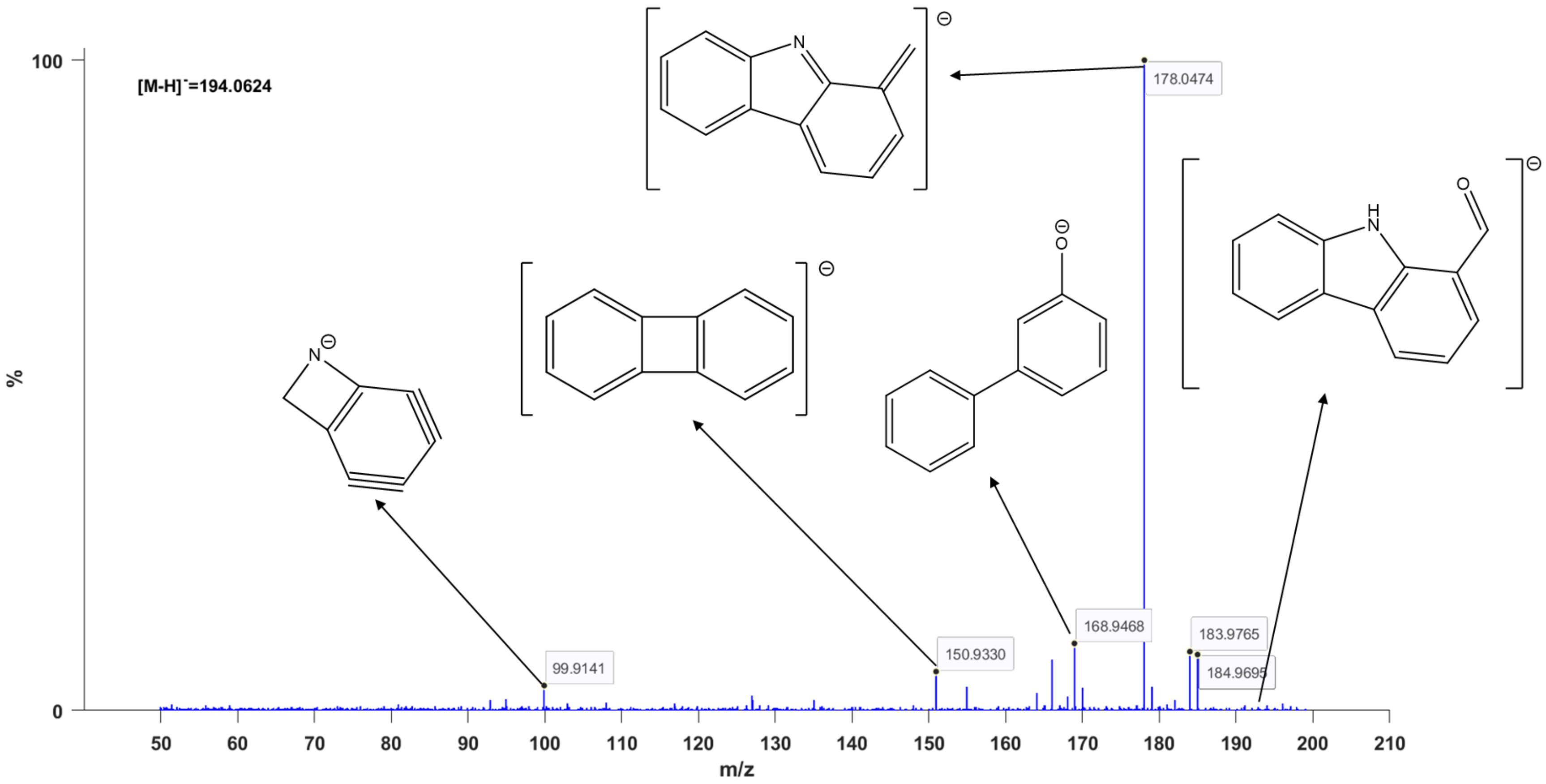

Figure S18. MS/MS spectrum of TP9 (ESI-).

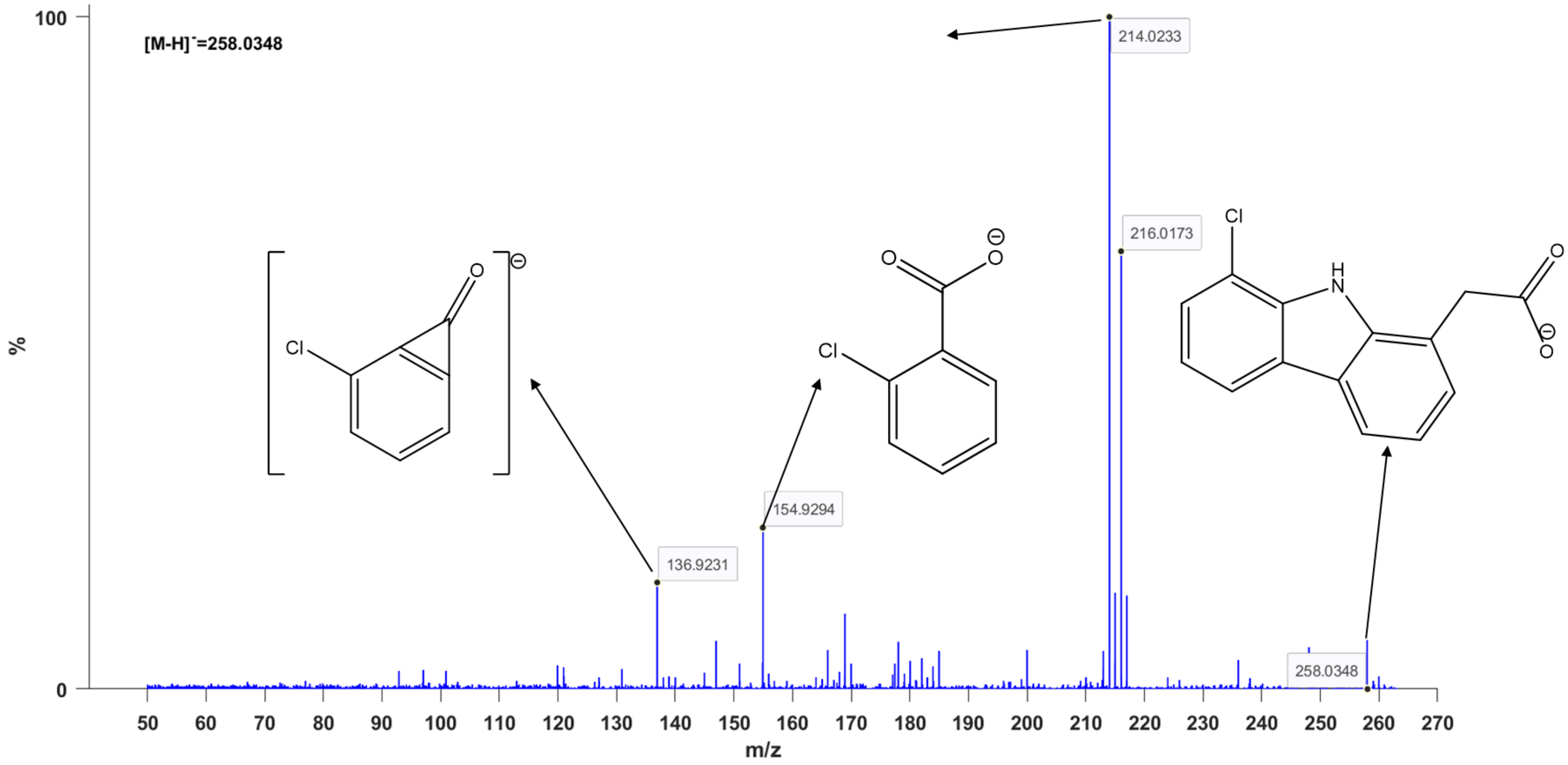

Figure S19. MS/MS spectrum of TP10 (ESI-).

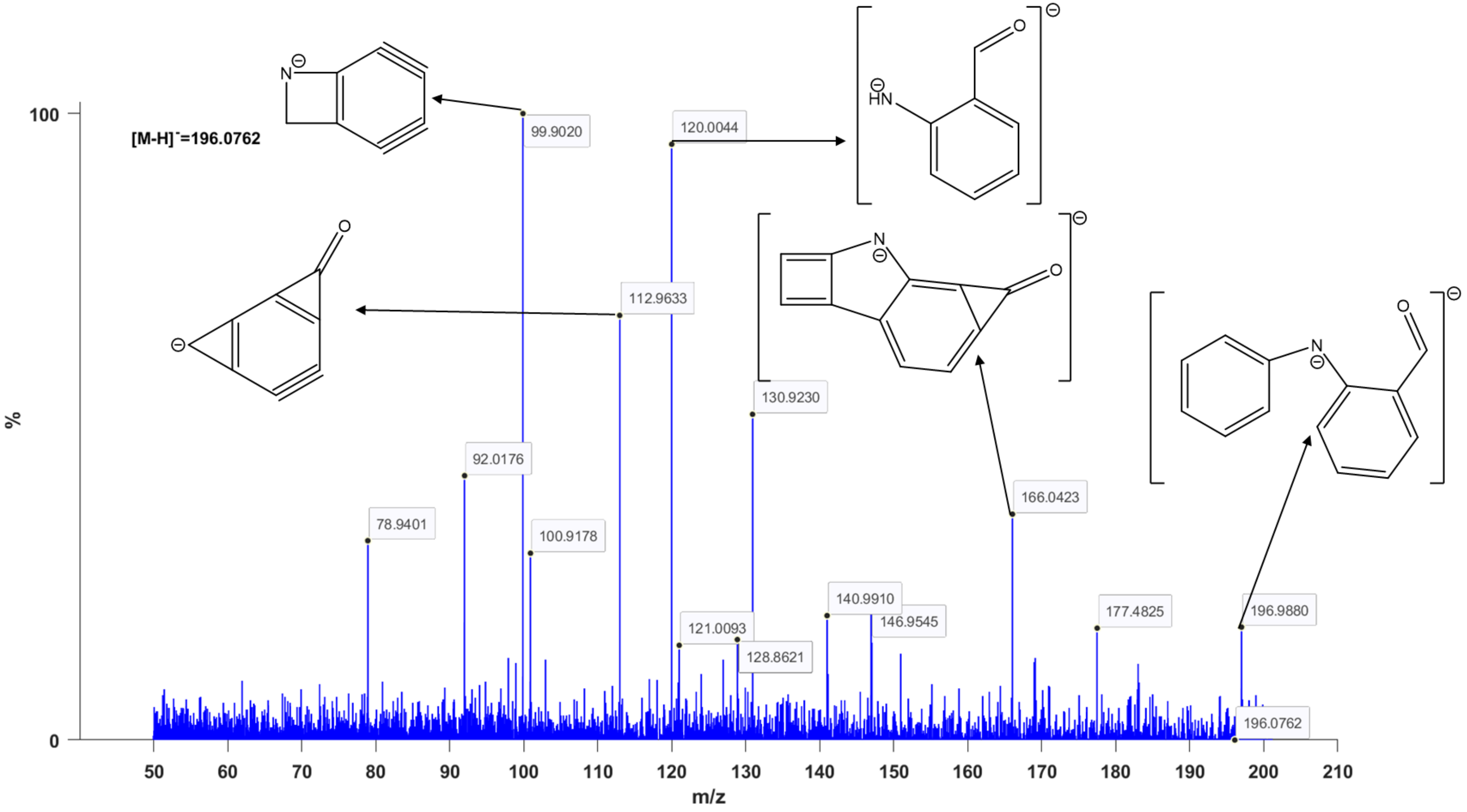

Figure S20. MS/MS spectrum of TP11 (ESI-).

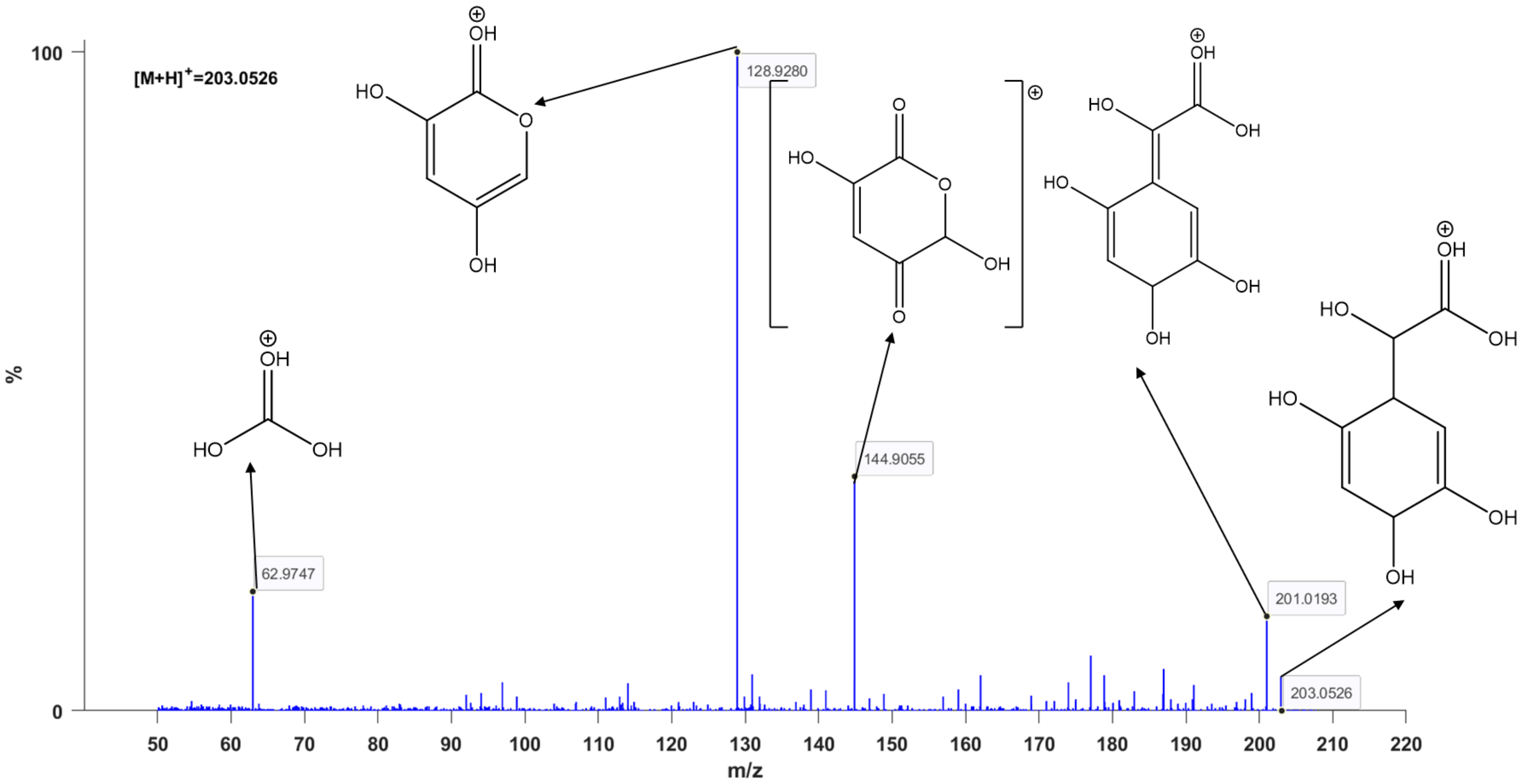

Figure S21. MS/MS spectrum of TP12 ESI+).

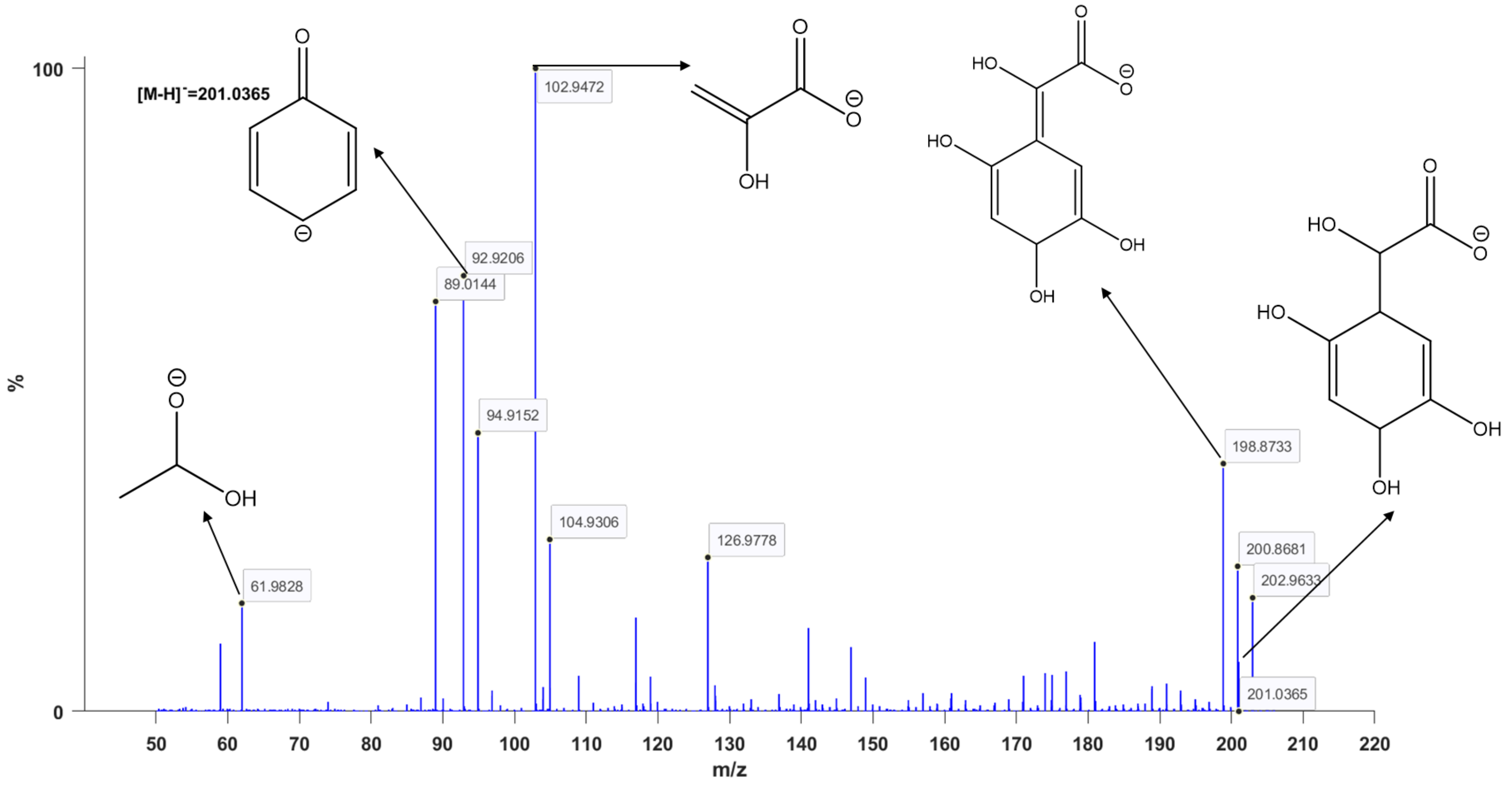

Figure S22. MS/MS spectrum of TP12 (ESI-).
